# Kobe project for the exploration of newer strategies to reduce the social burden of dementia: a study protocol of cohort and intervention studies

**DOI:** 10.1136/bmjopen-2021-050948

**Published:** 2021-06-17

**Authors:** Yoji Nagai, Shinsuke Kojima, Hisatomo Kowa, Yasuji Yamamoto, Hiroyuki Kajita, Tohmi Osaki, Yasumasa Kakei, Kavita U Kothari, Ryoma Kayano

**Affiliations:** 1 Department of Clinical Research Facilitation Institute for Advancement of Clinical and Translational Science(iACT), Kyoto University Hospital, Kyoto, Kyoto, Japan; 2 Institute of Medical Research and Innovation, Translational Research Center for Medical Innovation, Foundation for Biomedical Research and Innovation, Kobe, Hyogo, Japan; 3 Division of Cognitive and Psychiatric Rehabilitation, Department of Rehabilitation Science, Kobe Univerity Graduate School of Health Sciences, Kobe, Hyogo, Japan; 4 Department of Biosignal Pathophysiology, Kobe University Graduate School of Medicine, Kobe, Hyogo, Japan; 5 Faculty of Rehabilitation, Kobe Gakuin University, Kobe, Hyogo, Japan; 6 Clinical and Translational Research Center, Kobe University Hospital, Kobe, Hyogo, Japan; 7 World Health Organization Centre for Health Development, Kobe, Hyogo, Japan

**Keywords:** dementia, epidemiology, neuropathology, health policy, protocols & guidelines

## Abstract

**Introduction:**

This research project addresses the lack of screening tools for the early detection of high-risk individuals for long-term care, through four individual studies.

Study 1 investigates the predictive ability of the ‘Kihon Check List’, study 2 the ‘Cognitive Function instrument’ and EuroQol-5 Dimension (EQ-5D) and study 3 the ‘Cognitive Function instrument’ and EQ-5D as well as the ‘Frail Kenshin’ health check-up, for incident long-term care certification over a follow-up period of up to 4 years. This is the first large prospective study to evaluate the predictive ability of these tools for the outcome measure long-term care certification. The last subsection of this project study four aims to explore a mixed methods intervention for delaying the need for long-term care. This section is purely exploratory, looking for clues for further studies.

**Methods and analysis:**

Baseline data have been collected through local government programs, as well as through postal self-reported questionnaires. The primary outcome variable for all studies is long-term care certification data. Statistical analysis will be carried out using Kaplan-Meier, Multiple Cox regression as well as logistic regression.

**Conclusion:**

This project hopes to identify tools effective in predicting long-term care need. This will enable identification of citizens that are of higher risk for long-term care in the near future. This subset of high-risk individuals can in the future be addressed for extra support/intervention.

**Ethics and dissemination:**

All studies have been approved by respective institutional ethical committees and the WHO ethical committee ERC.0002899. In addition, all studies conform to the provisions of the Declaration of Helsinki and are conducted in accordance with Japan’s ‘Ethical Guidelines for Medical and Health Research Involving Human Subjects’. All findings will be disseminated at conferences and published in peer-reviewed journals.

**Trial registration number:**

UMIN000023283.

**Strengths and limitations of this study**

- This research project includes population based studies that allow for simple and reliable assessments of the risk for future long-term care need.
- This research project uses big data, which are collected by the local government (municipal office).
- The various baseline measures give a broad instrument to use for the prediction of long-term care needs concomitant with cognitive decline.
- The duration of follow-up of respective studies, which range from 2–4 years, is limited and further longer-term studies will be required.
- Study 4 has limitations, since it is a small sample and limited single-centre intervention study.

## Background and purpose

Due to high life expectancies and low birth rates, Japan has a rapidly ageing population. Since 2010, the proportion of population 65 years and older increased over 21%, making Japan a super-aged society.^1^ In 2019, this proportion reached 28% of the population.^2^ One of the major healthcare challenges is an increased need for long-term care (LTC). Dementia is one of the leading causes for care-requiring status, accounting for nearly 17% of individuals in LTC.^3^ Furthermore, the heart-breaking nature of the disease, accompanied with the significant impact on families and society, makes it a public health priority.^4^ Japan has the highest dementia prevalence (2.3% of the population) among Organisation for Economic Co-operation and Development (OECD) countries in 2017, and is projected to reach 3.8% by 2037.^5^

In spite of the urgent need to reduce the social burden, there are currently no therapeutic strategies to halt or slow the disease process.^6 7^ Lessons from prior studies recommend a focus on preclinical dementia and targeting of potential high-risk individuals who might benefit the most.^8^ In line with such recommendations, the focus of dementia research is shifting to earlier stages of cognitive decline, in the hope of mitigating or even reversing the pathophysiological processes.^9 10^ The achievements are still limited, in part due to the lack of efficient screening and evaluation methods for detecting higher risk individuals in society.

Under these circumstances, the Japanese Ministry of Health, Labour and Welfare (MHLW) issued a 5-year plan in 2013, for advancing measures against dementia, called ‘The Orange Plan’.^11^ In accordance with this plan, every municipality began to provide a variety of civil services in an attempt to tackle dementia, hoping to reduce the increasing social burden. For example, Kobe, the sixth largest city in Japan, initiated social participation programmes, aiming to prevent or mitigate cognitive decline, including the ‘Kihon Check List’ (KCL) programme, brain health school programme, and senior college programme. The effects of such services, however, have not been adequately evaluated due to the lack of appropriate evaluation metrics, and thus, improvements were hampered. Furthermore, LTC need data, is independently certified in the municipal office, with no link to dementia or other health promotion civil services.

The current project was designed to enable earlier identification of individuals at risk for dementia and consequently LTC. The first three studies use various cognition related questionnaires in an attempt to predict LTC need and identify individuals at higher risk. The last study is a small exploratory study which explores possible early interventions in these high-risk individuals to slow the progression of cognitive decline. This article introduces and outlines four individual studies included in this project.

## Methods

The current project comprises of four individual studies focusing on cognition-related parameters and LTC need certification in healthy community-dwelling citizens in Kobe city. Eligibility criteria of respective studies are independently defined, depending on the purpose of each study. Studies 1 and 4a were approved by the Ethics Committee in February 2017 and studies 2 and 3 in March 2017. All studies are currently ongoing.

### Evaluation and outcome measures

Questionnaires used in these studies are available in online supplemental files 1–5.

10.1136/bmjopen-2021-050948.supp1 Supplementary data

10.1136/bmjopen-2021-050948.supp2 Supplementary data

10.1136/bmjopen-2021-050948.supp3 Supplementary data

10.1136/bmjopen-2021-050948.supp4 Supplementary data

10.1136/bmjopen-2021-050948.supp5 Supplementary data

#### Kihon Check List

KCL is a self-reported frailty questionnaire designed by the MHLW, to identify frail elderly individuals at risk for LTC. The questionnaire used can be found in online supplemental file 1. It consists of a 25-item self-reported questionnaire and has been widely delivered to citizens from local governments across the nation from 2006 to 2016. It covers several domains of instrumental activities for daily living (ADLs), including general independence, physical strength, nutritional status, oral function, housebound, cognitive function, depression risk and general health status. In particular, the KCL includes three cognitive function-related questions—Do your family or your friends point out your memory loss? Do you make a call by looking up phone numbers? Do you find yourself not knowing today’s date?

#### Cognitive Function Instrument

Cognitive Function Instrument (CFI) is a screening tool to detect early changes of ADL due to cognitive decline.^12^ It was developed by the Alzheimer’s disease Cooperative Study over the last decade. The questionnaire used can be found in online supplemental file 2. CFI consists of 14 questions that are related to functional ability changes as a result of cognitive impairments. It is brief and easily administered at home through post, telephone or transmitted electronically. The CFI score is calculated by summing 1 point for ‘Yes’, 0 point for ‘No’ and 0.5 for ‘Maybe’. Although the CFI questionnaire includes ‘self’ and ‘partner’ versions, this project uses the ‘self’ version, because the current surveillance set up did not allow for both versions.

#### EuroQol-5 Dimension

EuroQol-5 Dimension (EQ-5D) is a standardised measure of health status developed by the EuroQol Group in order to provide a simple, generic measure of health for clinical and economic appraisal. It is applicable to a wide range of health conditions and treatments. The questionnaire used can be found in online supplemental file 3. EQ-5D is designed for self-completion and is suited for use in postal surveys, in clinics, and in face-to-face interviews. It is officially available in 171 languages and the official Japanese language version has been selected for the purpose of this study. The EuroQol Group provides two version of the EQ-5D with 3 or 5 dimensions, the latter of which (EQ-5D-5-level (5L)) was adopted in this project. The EQ-5D-5L includes five categories with five questions in each category, summing up to 25 questions. Based on the pattern of answers, the health status of an individual is given a single index value, ranging from zero to one, death to perfect health.

#### Frail Kenshin

The ‘Frail Kenshin’ check-up is an elderly citizen health check-up programme by Kobe city, which is offered to citizens covered by the ‘Kokumin Kenko Hoken’ insurance (Kokumin Kenko Hoken is the National Health Insurance system in Japan, it includes all Japanese citizens and long-term residents). The questionnaires used along with this check-up can be found in online supplemental files 4 and 5. It has physically accessed components as well as self-assessed questions related to frailty. It evaluates, motor function, nutrition, oral function, forgetfulness, mental health, chewing ability, dental health, swallowing ability, grip strength, height (cm), weight (kg), body mass index and more.

#### LTC certification

Under the national LTC Insurance Act, certification of LTC need has been implemented since April 2000. The main beneficiaries are elderly individuals aged 65 or over. LTC need certification is routinely conducted by each municipal office on an application basis from citizens. LTC need status is categorised into eight levels (no need for support or care: 0, support level: 1 or 2, care level: 1–5). In the current project, certified care levels from 1 (partial assistance) to 5 (total assistance with disabled communication) were defined as to be in need of LTC.

So far, identification of individuals at higher risk of dementia has been hampered, largely because of the lack of convenient screening methods. Appropriate evaluation measures play a crucial role for the success of this project. For example, measures for baseline evaluation need to be easy to use, easy to answer, and quantitative. Also, measures for outcome evaluation need to be fitted for purpose, objective and must be readily observable in local governmental services. As measures to meet such requirements, KCL, CFI, EQ-5D and LTC certification have been chosen as some of the baseline and/or outcome evaluation tools. KCL has been proven to be a reliable indicator of frailty,^13^ and is considered advantageous for a population-based approach.^14^ EQ-5D is an established standard measure for health-related QOL, and is widely used around the world. In contrast, CFI is a relatively new measure, which could be used in large epidemiological and clinical studies with minimal in-person contact. In a previous study by Amariglio *et al*,^12^ the CFI score was gradually worse in individuals who developed dementia than in those that did not, over a period of 4 years. Although their finding does not directly demonstrate the utility of CFI for risk assessment of dementia, it suggests value to be tested in larger prospective studies. However, it should be noted that CFI is a measure for the change of ADL due to cognitive decline, and not a direct measure for cognitive function per se. Thus, the results to be obtained need to be interpreted with caution, yet it is a potentially useful tool for the risk stratification of LTC need in the community.

### Outline of studies included in the project

The four studies are briefly explained below and summarised in table 1.

**Table 1:** Summary of studies

	Study 1	Study 2	Study 3	Study 4
Target population for recruitment	General Population (80 000)	Citizens with Possible Cognitive Decline (4131)	‘Frail Check’ Participants (4800)	Brain health school programme participants (80)
Sample population	50 155	2385	2705	57
Baseline evaluation	KCL	CFI, EQ-5D, GDS	CFI, EQ-5D, General Frailty Measures	MMSE, EQ-5D CFI, GDS, etc
Primary outcome	LTC Need	LTC Need	LTC Need	MMSE
Secondary outcome	LTC Need Level	CFI, EQ-5D	LTC Need Level	EQ-5D, CFI, Five Cog, etc
Intervention	None	None	None	Intensive Cognitive Training
Study period	4–5 years	2 years	2 years	30 months

CFI, Cognitive Function Instrument; GDS, Geriatric Depression Scale; KCL, Kihon Check List; LTC, long-term care; MMSE, Mini Mental State Examination.

#### Study 1: elderly cohort study for LTC need risk stratification

This study is a prospective cohort study that investigates the predictive ability of the KCL and the cognitive domain therein for incident LTC certification. The target group was approximately 80 000 elderly Kobe citizens to whom KCL was sent in 2015.

Inclusion: The general population will be eligible for inclusion if they are aged 70 years or older, responded to the KCL of 2015, and were not LTC need certified at the time of the KCL data collection.

A total of 77 900 target population with an estimated response rate of 60%, thus, 50 000 people are expected to participate in the study. The primary outcome is to be certified for LTC need. The secondary endpoints include levels (1–5) and causes (dementia or physical disability) of LTC need. The KCL data are collected via postal mail survey by the Kobe municipal office, which will be merged with LTC need certification data. The follow-up duration is calculated from the date of first response to KCL to the date of LTC need certification until a cut-off date of November 2019 (4 years).

For the statistical analysis, the incidence of LTC need at 1, 2, 3 and 4 years after KCL distribution will be estimated using the Kaplan-Meier method. For the estimation, data for all eligible citizens will be included in the time-to-event analysis. The follow-up duration is calculated from the date of first response to KCL to the date of LTC need certification up until a cut-off date of November 2019.

Subsequently, multivariate Cox regression analysis will be performed using the stepwise variable selection method to narrow down independent variables and to estimate the associated HR. Multivariate Cox Regression Analysis will be performed for 27 variables; age, sex and 25 questions of the KCL. Statistical analysis will be carried out using the SAS V.9.3 (SAS Institute). The significance threshold is set at p<0.05.

#### Study 2: quantifying the risk of LTC need in citizens with possible cognitive decline

This is a prospective cohort study which examines the predictive ability of CFI and EQ-5D questionnaires, for LTC certification, in elderly citizens at higher risk of dementia. The sample population is a subgroup population of those that responded to the KCL of 2015 (a subgroup of study 1). The target population are those that responded with unfavourable answers to either of the three cognitive domain questions in the KCL from the 2015 survey. The major inclusion criteria are citizens who are above or equal to 70 years old at the time of KCL survey, had at least one unfavourable answer to the cognitive domain questions in the KCL, those that responded to CFI and EQ-5D questionnaires in 2017 and 2019, and are not LTC certified. The target population is 4131 citizens which were sent CFI, and EQ-5D in 2017. Out of which 2981 citizens responded. In 2019, this same cohort were sent the CFI and EQ-5D, out of which 2444 citizens replied. After discounting for uncompleted forms, 2292 formed the sample for this study. The primary endpoint is the occurrence of LTC need certification after 2 years, and the secondary endpoints are the change in CFI and EQ-5D during the same period. After matching for LTC certification, statistical analysis will be carried out with the use of proportional Hazard model. Relative risk for future LTC need and death is estimated in relation to the baseline CFI and EQ-5D. The relationship between the changes in such parameters and LTC need will also explored. Repeated measure analysis of variance (ANOVA) or mixed effect model will be used to examine the changes of CFI and EQ-5D, in relation to occurrence of LTC need.

#### Study 3: quantifying the risk of LTC need in generally healthier citizens

This study explores the relationship of CFI, EQ-5D and other frailty-related parameters, with the risk of future LTC need within 3 years. It will search for other prognostic factors that may predict the progression to LTC. The target population was Kobe residents who turned 65 years old in 2017 or 2018 and who underwent ‘Frail Kenshin’. ‘Frail Kenshin’ is an elderly citizen health check-up programme by Kobe city, which is offered to citizens covered by the ‘Kokumin Kenko Hoken’ insurance (Kokumin Kenko Hoken is the National Health Insurance system in Japan, it includes all Japanese citizens and long-term residents). ‘Kokumin Kenko Hoken’ insurance covered citizens who turned 65 in 2017 or 2018 were 28 800 citizens, out of which 2705 people appeared for the ‘Frail Kenshin’ check-up of 2017 and 2018 and formed the sample for this study. Thus, the target citizens are younger than study 1 and 2, and not known to be at risk for dementia. They are also likely to be health-conscious citizens since they appeared for a voluntary health check-up. The major inclusion criteria are those who turn or turned 65 years old during 2017 or 2018, underwent ‘Frail Kenshin’ check-up and were not LTC certified at the time of the check-up. The primary endpoint is the occurrence of LTC need, and the secondary endpoints include LTC care levels (1–5). CFI and EQ-5D questionnaires were carried out at the venue of check-up, concomitant with other frailty checks, and the completed questionnaires were collected and checked by the person in charge.

First the incidence of LTC over the study period will be calculated using LTC care level 1 or greater. The statistical methods to be used are logistic regression model (estimated by least absolute shrinkage and selection operator: LASSO) with the presence and absence of LTC care as the dependent variable with CFI Self score, EQ-5D scores and other ‘Frail Kenshin’ check-up results as explanatory variables. A formula and Receiver Operatorating Characteristic (ROC) curve will be estimated. Multiple regression will also be carried out for continuous variables used in the ‘Frail Kenshin’ health check-up. In addition, age and gender are included in the model as explanatory variables in all analysis. In the case that the significance level of the above-mentioned test is p<0.05 on both sides, the multiplicity of the tests is not considered an issue. SAS V.9.4 and R V.3.6 are used for the analysis.

#### Study 4: study on effect of multimodal dementia prevention programme in community-dwelling elderly

This is a parallel exploratory intervention study, to explore the effects of multimodal interventions for slowing the process of cognitive decline. The target group were approximately 80 citizens over 70 years old who participated in a dementia prevention education programme which was held monthly by Kobe city in 2016. Individuals who agreed to participate in this study are randomly divided into two groups. Both groups undergo an intensive training programme (education, cognitive training and exercise) once a week for a total of 10 weeks and are monitored every 6 months for up to 30 months after recruitment. During the follow-up period, the intervention group receives a booster training once every 3 months, whereas the control group does not. The endpoints include changes in Mini Mental State Examination, CFI, EQ-5D, Five Cog, Timed Up & Go test, Physical Activity Scale for the Elderly and Geriatric Depression Scale. In the ANOVA between the three groups, effect size was set at 0.25, α=0.05 and power=0.80, the necessary data number will be approximately 50 persons for each group. Since this is a long-term longitudinal study, the number of subjects is set at a higher 100 persons, which is about double the necessary number (for statistical significance) as a cautionary measure for drop outs and missing data. The longitudinal change of the evaluation results in each group is analysed using repeated ANOVA and mixed effects model. The amount of change in the evaluation result between groups is compared by multiple comparison tests: the paired t-test or Wilcoxon test.

## Discussion

In a prior study (Kojima *et al*), an analysis of 182 099 Kobe citizens showed, cognitively unfavourable answers in KCL were found to be associated with the incidence of LTC need, independent of age, sex and other items on KCL.^15^ Furthermore, as the number of unfavourable answers to the cognitive domain increased from 0 to 1, 2 and 3, the incidence of LTC need progressively increased from 3.5% to 6.4%, 12.6% and 29.6% after 3 years. This finding suggests a potential utility for KCL in the risk stratification of future LTC need. However, because this was a retrospective analysis on pooled data, the causal relationship between cognitively unfavourable answers and LTC need could not be validated. The current project will test the predictive ability of the KCL as well as other tools such as CFI, EQ-5D and ‘Frail Kenshin’ check-up in relation to LTC need.

Each of the four studies in this project focuses on citizens at a different level of dementia risk (study 1: general population, study 2: higher risk population, study 3: lower risk population, study 4: higher risk individuals willing to participate in the training programme, see table 1).

### Strengths and weakness of the study

**Figure 1:**
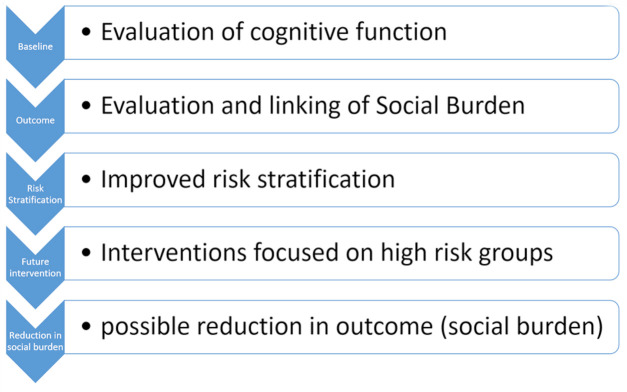
Project concept. This figure depicts the concept of this project. If baseline and outcome evaluation, can we repeated and incorporated into government practice, a foundation for the focus on high-risk individuals and possible reduction in LTC (social burden) can be realised. LTC, long-term care.

A limitation of the study is that the follow-up study period is relatively short, due to the contract duration by the funder. However, if this evaluation system is incorporated into local governmental systems in the future, follow-up periods can be virtually unlimited, and baseline and outcome evaluation would be repeated (figure 1). Through this process a quantitative feedback mechanism could be created forming the foundation to reduce the social burden of dementia, which further on, could also be extended to address other public health problem areas.^16 17^ Additionally, study 4 is a highly exploratory study with limited sample size. This study hopes to find what is feasible and possibly provide clues for improving the social burden of LTC need, which can then be tested in future studies.

#### Meaning of study

By the completion of study 1, 2 and 3, risk stratification of earlier cognitive decline for future LTC need will be improved on a population basis. Particularly, if individuals at risk for future dementia could be extracted, social efforts could be directed to such individuals who may receive a greater benefit, allowing for a more efficient allocation of limited public resources. Also, given the failure of many clinical trials for dementia drug development, the system constructed by this project could help prepare ‘trial-ready cohorts’, to increase efficiency of recruiting eligible participants.

In summary, this project hopes to show a risk prediction and early intervention model linked to the outcome measurement, LTC. If continuously implemented, it can be a foundation for addressing the burden of dementia earlier. The final hope of this project is ‘not to be demented’, but if demented, ‘not to be in need of care’.

### Patient and public involvement

No Patient Involved.

## Ethics and dissemination

This study complies with the Declaration of Helsinki. These studies were approved by the Ethics Review Committee of the WHO (ERC.0002899) as well as respective organisational ERC’s, and the Ethics Review Committee of the Kobe Municipal office. In addition, the study conforms to the provisions of the Declaration of Helsinki and are conducted in accordance with Japan’s “Ethical Guidelines for Medical and Health Research Involving Human Subjects.

The researchers plan to disseminate results at conferences and in peer-reviewed journals.

## Supplementary Material

Reviewer comments

Author's manuscript
