# Supplementary material for: Kobe project for the exploration of newer strategies to reduce the social burden of dementia: a study protocol of cohort and intervention studies
Source: BMJ Open. 2021 Jun 17;11(6):e050948. doi: 10.1136/bmjopen-2021-050948 (PMC8215256; doi:10.1136/bmjopen-2021-050948)
Supplement: Supplementary data [file bmjopen-2021-050948supp001.pdf]

Kihon Checklist

No.	Questions	Answer	
1	Do you go out by bus or train by yourself?	□0. YES	□1. NO
2	Do you go shopping to buy daily necessities by yourself?	□0. YES	□1. NO
3	Do you manage your own deposits and savings at the bank?	□0. YES	□1. NO
4	Do you sometimes visit your friends?	□0. YES	□1. NO
5	Do you turn to your family or friends for advice?	□0. YES	□1. NO
6	Do you normally climb stairs without using handrail or wall for support?	□0. YES	□1. NO
7	Do you normally stand up from a chair without any aids?	□0. YES	□1. NO
8	Do you normally walk continuously for 15 minutes?	□0. YES	□1. NO
9	Have you experienced a fall in the past year?	□1. YES	□0. NO
10	Do you have a fear of falling while walking?	□1. YES	□0. NO
11	Have you lost 2kg or more in the past 6 months?	□1. YES	□0. NO
12	Height: cm, Weight: kg, BMI: kg/m^2 If BMI is less than 18.5, this item is scored.	□1. YES	□0. NO
13	Do you have any difficulties eating tough foods compared to 6 months ago?	□1. YES	□0. NO
14	Have you choked on your tea or soup recently?	□1. YES	□0. NO
15	Do you often experience having a dry mouth?	□1. YES	□0. NO
16	Do you go out at least once a week?	□0. YES	□1. NO
17	Do you go out less frequently compared to last year?	□1. YES	□0. NO
18	Do your family or your friends point out your memory loss?<br>e.g."You ask the same question over and over again."	□1. YES	□0. NO
19	Do you make a call by looking up phone numbers?	□0. YES	□1. NO
20	Do you find yourself not knowing today’s date?	□1. YES	□0. NO
21	In the last 2 weeks have you felt a lack of fulfillment in your daily life?	□1. YES	□0. NO
22	In the last 2 weeks have you felt a lack of joy when doing the things you used to enjoy?	□1. YES	□0. NO
23	In the last 2 weeks have you felt difficulty in doing what you could do easily before?	□1. YES	□0. NO
24	In the last 2 weeks have you felt helpless?	□1. YES	□0. NO
25	In the last 2 weeks have you felt tired without a reason?	□1. YES	□0. NO

(Working Group on Frailty in JGS)
