# Supplementary material for: Kobe project for the exploration of newer strategies to reduce the social burden of dementia: a study protocol of cohort and intervention studies
Source: BMJ Open. 2021 Jun 17;11(6):e050948. doi: 10.1136/bmjopen-2021-050948 (PMC8215256; doi:10.1136/bmjopen-2021-050948)
Supplement: Supplementary data [file bmjopen-2021-050948supp002.pdf]

Site: ______________

Participant Number: ______________

Individual Overseeing Completion:

Initials: _______ Date: ___________

Cognitive Function Index
Participant

Instructions: Please complete this form independently, without consulting anyone. Answer all questions with reference to one year ago.

1. Compared to one year ago, do you feel that your memory has declined substantially?
   ☐ **Yes** ☐ **No** ☐ **Maybe**
2. Do others tell you that you tend to repeat question over and over?
   ☐ **Yes** ☐ **No** ☐ **Maybe**
3. Have you been misplacing things more often?
   ☐ **Yes** ☐ **No** ☐ **Maybe**
4. Compared to one year ago, do you find that you are relying more on written reminders (e.g., shopping lists, calendars)?
   ☐ **Yes** ☐ **No** ☐ **Maybe**
5. Do you need more help from others to remember appointments, family occasions or holidays?
   ☐ **Yes** ☐ **No** ☐ **Maybe**
6. Do you have more trouble recalling names, finding the right word, or completing sentences?
   ☐ **Yes** ☐ **No** ☐ **Maybe**
7. Do you have more trouble driving (e.g., do you drive more slowly or too fast, have more trouble at night, tend to get lost, have accidents)?
   ☐ **Yes** ☐ **No** ☐ **Maybe** ☐ **Does Not Apply**
8. Compared to one year ago, do you have more difficulty managing money (e.g., paying bills, calculating change, completing tax forms)?
   ☐ **Yes** ☐ **No** ☐ **Maybe** ☐ **Does Not Apply**
9. Are you less involved in social activities?
   ☐ **Yes** ☐ **No** ☐ **Maybe**
10. Has your work performance (paid or volunteer) declined significantly, compared to one year ago?
   ☐ **Yes** ☐ **No** ☐ **Maybe** ☐ **Does Not Apply**

Nagai Y, *et al. BMJ Open* 2021; 11:e050948. doi: 10.1136/bmjopen-2021-050948

Site: ______________

Participant Number: ______________

Cognitive Function Index

Participant

Individual Overseeing Completion:

Initials: _______ Date: ___________

11. Do you have more trouble following the news or the plots of books, movies, or TV shows?

☐ **Yes** ☐ **No** ☐ **Maybe**

12. Are there any activities (e.g., hobbies such as card games, crafts) that are substantially more difficult for you now compared to one year ago?

☐ **Yes** ☐ **No** ☐ **Maybe**

13. Are you more likely to be come disoriented, or get lost, for example when traveling to another city?

☐ **Yes** ☐ **No** ☐ **Maybe**

14. Compared to one year ago, do you have more difficulty using household appliances (such as washing machine or microwave) or electronic devices (such as computer or cell phone)?

☐ **Yes** ☐ **No** ☐ **Maybe**

15. In the past year, have you seen a doctor about memory concerns?

☐ **Yes** ☐ **No**

To be completed by study staff:

**Method of Collection**	**Comments:**
___ In-person/Self<br>___ By phone/Interview	______________________<br>______________________<br>______________________
