# Supplementary material for: Kobe project for the exploration of newer strategies to reduce the social burden of dementia: a study protocol of cohort and intervention studies
Source: BMJ Open. 2021 Jun 17;11(6):e050948. doi: 10.1136/bmjopen-2021-050948 (PMC8215256; doi:10.1136/bmjopen-2021-050948)
Supplement: Supplementary data [file bmjopen-2021-050948supp003.pdf]

健康アンケート

日本用日本語版

(Japanese version for Japan)

Japan (Japanese) © 2009 EuroQol Group. EQ-5D™ is a trade mark of the EuroQol Group

各項目において、あなたの**今日の**健康状態を最もよく表している四角(❑)**1 つ**に✓印をつけてください。

移動の程度

歩き回るのに問題はない ❑

歩き回るのに少し問題がある ❑

歩き回るのに中程度の問題がある ❑

歩き回るのにかなり問題がある ❑

歩き回ることができない ❑

身の回りの管理

自分で身体を洗ったり着替えをするのに問題はない ❑

自分で身体を洗ったり着替えをするのに少し問題がある ❑

自分で身体を洗ったり着替えをするのに中程度の問題がある ❑

自分で身体を洗ったり着替えをするのにかなり問題がある ❑

自分で身体を洗ったり着替えをすることができない ❑

ふだんの活動 *（例：仕事、勉強、家事、家族･余暇活動）*

ふだんの活動を行うのに問題はない ❑

ふだんの活動を行うのに少し問題がある ❑

ふだんの活動を行うのに中程度の問題がある ❑

ふだんの活動を行うのにかなり問題がある ❑

ふだんの活動を行うことができない ❑

痛み ／ 不快感

痛みや不快感はない ❑

少し痛みや不快感がある ❑

中程度の痛みや不快感がある ❑

かなりの痛みや不快感がある ❑

極度の痛みや不快感がある ❑

不安 ／ ふさぎ込み

不安でもふさぎ込んでもいない ❑

少し不安あるいはふさぎ込んでいる ❑

中程度に不安あるいはふさぎ込んでいる ❑

かなり不安あるいはふさぎ込んでいる ❑

極度に不安あるいはふさぎ込んでいる ❑

Japan (Japanese) © 2009 EuroQol Group. EQ-5D™ is a trade mark of the EuroQol Group

- あなたの**今日の**健康状態がどのくらい良いか悪いかを教えてください。
- このものさしには **0** から **100** までの目盛がふってあります。
- **100** はあなたの想像できる最も良い健康状態を、

  0 はあなたの想像できる最も悪い健康状態を表しています。
- **今日の**健康状態がどのくらい良いか悪いかを、このものさし上に**×**印をつけて表してください。
- ものさし上に**×**印をつけたところの目盛を下の四角に記入してください。

あなたの今日の健康状態 ＝ ☐

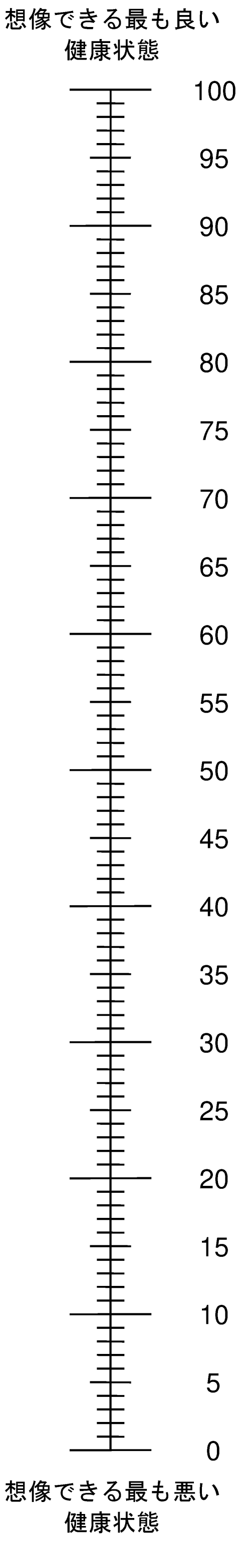

Japan (Japanese) © 2009 EuroQol Group. EQ-5D™ is a trade mark of the EuroQol Group
