# Supplementary material for: Kobe project for the exploration of newer strategies to reduce the social burden of dementia: a study protocol of cohort and intervention studies
Source: BMJ Open. 2021 Jun 17;11(6):e050948. doi: 10.1136/bmjopen-2021-050948 (PMC8215256; doi:10.1136/bmjopen-2021-050948)
Supplement: Supplementary data [file bmjopen-2021-050948supp004.pdf]

神戸市　「フレイル健診」試行実施　計測（運動・筋力、咀嚼力、嚥下）結果記入票

下記の太枠内をご記入ください。

氏名　　　　　　　　男・女	生年月日　　　　　昭和　　年　　月　　日　（　　）才<br>神戸市国民健康保険 被保険者番号：受診券・健康保険証に記載あり（　　　　　　）
１．【ふくらはぎ周囲長】<br>（　　　　　）ｃｍ<br>男性は 34ｃｍ未満、女性は 32ｃｍ未満の場合は、要注意です。	２．【指輪っかテスト】　（　太くて囲めない　・　ちょうど囲める　・　隙間ができる　）<br>＊「指輪っかテスト」は、東京大学社会総合研究機構で考案されたものです。<br>「隙間ができる」に当てはまる場合には、筋肉や筋量が減っている可能性があります。<br>ご自分で筋肉の低下を測る目安となります。筋力の衰えが気になる時に測定してみましょう。

３．【かむ力判定ガム】　　緑　・　黄　・　桜色　・　濃い桜色　・　紅色

＜咀嚼力＞　弱い　→　強い

弱い　　やや弱い　　普通　　やや強い　　強い

一口（ひとくち）30 回よくかんで食べること（カミング３０）は、全身の健康や認知症予防に大きな効果があります。かめない場合は、かかりつけ歯科医を受診しましょう。

４．【だ液ゴックンテスト】　30 秒の間に唾液を飲み込んだ回数　（2 回以下・3 回以上）　3 回以上飲み込めれば、問題はありません。

結果		５．　握力　右手（　　　）ｋｇ 左手（　　　）ｋｇ ＊握力が強い方の手の握力					６．　立ち上り　（　　　）回／１５秒				
性別・年代		弱い	やや弱い	ふつう	やや強い	強い	遅い	やや遅い	ふつう	やや早い	早い
男性	65－69	～24kg	24.1～31.0	31.1～34.0	34.1～43.0	43.1～	～4回	5～7	8	9～11	12以上
	70－74	～22kg	22.1～29.0	29.1～32.0	32.1～40.0	40.1～	～ 4回	5～6	7	8～10	11以上
女性	65－69	～15kg	15.1～19.0	19.1～21.0	21.1～27.0	27.1～	～ 4回	5～7	8	9～11	12以上
	70－74	～14kg	14.1～18.0	18.1～20.0	20.1～26.0	26.1～	～ 4回	5～6	7	8～10	11以上
取り入れたい運動		握力は全身の筋力の状況と比例していて全身の体力の把握に役立ちます。「弱い」、「やや弱い」の方は生活に体操や筋力トレーニングを取り入れましょう。筋力が向上すると転倒予防になり、いろいろな動作がしやすくなります。					立ち上がり動作は足腰の筋力の強さや持久力・スタミナの目安になります。「遅い」、「やや遅い」の方は、散歩・ジョギング・ハイキングなどを取り入れましょう。坂道や階段の昇り降りが楽になり、楽に歩ける距離が長くなります。				
