# Supplementary material for: Kobe project for the exploration of newer strategies to reduce the social burden of dementia: a study protocol of cohort and intervention studies
Source: BMJ Open. 2021 Jun 17;11(6):e050948. doi: 10.1136/bmjopen-2021-050948 (PMC8215256; doi:10.1136/bmjopen-2021-050948)
Supplement: Supplementary data [file bmjopen-2021-050948supp005.pdf]

氏名 男・女	電話番号
〒 住所 区	
生年月日 昭和 年 月 日 （ ）才	神戸市国民健康保険 被保険者番号 ＊受診券・健康保険証に記載あり （ ）

神戸市「フレイル健診」試行実施　受診票・質問票

この健診結果は、受診者本人に対して通知するとともに、神戸市において保存し、必要に応じて介護予防のためのご案内や、個人が特定されない方法で統計・調査研究などに活用しますので、ご了承の上、ご受診願います。

受診日　年　月　日　受診会場名

以下の太枠内を、ご記入ください。

1	この１年間に特定健診などの健診を受けましたか。	はい	いいえ

日常生活活動・もの忘れチェック

２．下記の質問にお答えください。

①	バスや電車で一人で外出していますか。	はい	いいえ
②	日用品の買い物をしていますか。	はい	いいえ
③	預貯金の出し入れをしていますか。	はい	いいえ
④	友人の家を訪ねていますか。	はい	いいえ
⑤	家族や友人の相談にのっていますか。	はい	いいえ
⑥	週に１回以上は外出していますか。	はい	いいえ
⑦	昨年と比べて外出の回数が減っていますか。	はい	いいえ
⑧	周りの人から「いつも同じ事を聞く」などのもの忘れがあると言われますか。	はい	いいえ
⑨	自分で電話番号を調べて、電話をかけることをしていますか。	はい	いいえ
⑩	今日が何月何日かわからない時がありますか。	はい	いいえ

運動機能チェック

３．下記の質問にお答えください。

①	階段を手すりや壁をつたわらずに昇っていますか。	はい	いいえ
②	椅子に座った状態から何もつかまらずに立ち上がっていますか。	はい	いいえ
③	15 分続けて歩いていますか。	はい	いいえ
④	この１年間に転んだことがありますか。	はい	いいえ
⑤	転倒に対する不安は大きいですか。	はい	いいえ

栄養状態チェック

４．下記の質問にお答えください。

①	６ヶ月間で２〜３ｋｇ以上の体重減少がありましたか。	はい	いいえ
②	身長 ． ｍ 体重 ｋｇ	―	―

口腔機能チェック

５．現在、ご自身の歯は何本残っていますか。当てはまる本数に〇を付けてください。さし歯や金属をかぶせた歯は自分の歯に含めます。成人の歯の総本数は、親知らずを含めて 32 本です。

①．０本	②．1〜４本	③．5〜９本	④．10〜19 本	⑤．20 本以上

６．次の ２５種類の食品について、噛めるものに〇、噛みづらいものに△、噛めないものに×をつけてください。

①バナナ		②茹でたキャベツ		③煮たにんじん		④煮たさといも	
⑤煮たたまねぎ		⑥いちご		⑦ハム		⑧かまぼこ	
⑨佃煮昆布		⑩こんにゃく		⑪鳥のからあげ		⑫ローストチキン	
⑬リンゴ		⑭なすの漬物		⑮生キャベツ		⑯焼いた豚肉	
⑰大根の漬物		⑱あられ		⑲ピーナッツ		⑳いか刺身	
㉑生のにんじん		㉒酢だこ		㉓するめ		㉔干しいも	
㉕かた焼きせんべい							
「〇」 個			「△」 個			「×」 個	

７．下記の質問にお答えください。

①	半年前に比べて固いものが食べにくくなりましたか。	はい	いいえ
②	お茶や汁物等でむせることがありますか。	はい	いいえ
③	口の渇きが気になりますか。	はい	いいえ

こころの健康チェック

８．下記の質問にお答えください。

①	（ここ２週間）毎日の生活に充実感がない。	はい	いいえ
②	（ここ２週間）これまで楽しんでやれていたことが楽しめなくなった。	はい	いいえ
③	（ここ２週間)以前は楽にできていたことが今はおっくうに感じられる。	はい	いいえ
④	（ここ２週間）自分が役に立つ人間だとは思えない。	はい	いいえ
⑤	（ここ２週間）わけもなく疲れたような感じがする。	はい	いいえ

実施機関処理欄
