# Supplementary material for: Kobe project for the exploration of newer strategies to reduce the social burden of dementia: a study protocol of cohort and intervention studies
Source: BMJ Open. 2021 Jun 17;11(6):e050948. doi: 10.1136/bmjopen-2021-050948 (PMC8215256; doi:10.1136/bmjopen-2021-050948)
Supplement: Author's manuscript [file bmjopen-2021-050948.draft_revisions.pdf]

BMJ Open is committed to open peer review. As part of this commitment we make the peer review history of every article we publish publicly available.

When an article is published we post the peer reviewers' comments and the authors' responses online. We also post the versions of the paper that were used during peer review. These are the versions that the peer review comments apply to.

The versions of the paper that follow are the versions that were submitted during the peer review process. They are not the versions of record or the final published versions. They should not be cited or distributed as the published version of this manuscript.

BMJ Open is an open access journal and the full, final, typeset and author-corrected version of record of the manuscript is available on our site with no access controls, subscription charges or pay-per-view fees (http://bmjopen.bmj.com).

If you have any questions on BMJ Open's open peer review process please email info.bmjopen@bmj.com

BMJ Open

Kobe Project for the Exploration of Newer Strategies to Reduce the Social Burden of Dementia: cohort and intervention studies

Journal:	*BMJ Open*
Manuscript ID	bmjopen-2021-050948
Article Type:	Protocol
Date Submitted by the Author:	04-Mar-2021
Complete List of Authors:	Nagai, Yoji; Kobe University Hospital, Clinical & Translational Research Center<br>Kojima, Shinsuke; Translational Research Center for Medical Innovation, Foundation for Biomedical Research and Innovation, Institute of Medical Research and Innovation<br>Kowa, Hisatomo; Kobe Univerity Graduate School of Health Sciences, Division of Cognitive and Psychiatric Rehabilitation, Department of Rehabilitation Science<br>Yamamoto, Yasuji; Kobe University Graduate School of Medicine School of Medicine, Department of Biosignal Pathophysiology<br>Kajita, Hiroyuki ; Kobe Gakuin University, Faculty of Rehabilitation<br>Osaki, Tohmi; Kobe Gakuin University Faculty of Rehabilitation Graduate School of Rehabilitation, Faculty of Rehabilitation<br>Kakei, Yasumasa; Kobe University Hospital, Clinical & Translational Research Center<br>Kothari, Kavita; Kobe University Hospital, Clinical & Translational Research Center<br>Kayano, Ryoma ; WHO Kobe Center
Keywords:	Dementia < NEUROLOGY, EPIDEMIOLOGY, Neuropathology < NEUROLOGY, Health policy < HEALTH SERVICES ADMINISTRATION & MANAGEMENT, Protocols & guidelines < HEALTH SERVICES ADMINISTRATION & MANAGEMENT

SCHOLARONE™
Manuscripts

I, the Submitting Author has the right to grant and does grant on behalf of all authors of the Work (as defined in the below author licence), an exclusive licence and/or a non-exclusive licence for contributions from authors who are: i) UK Crown employees; ii) where BMJ has agreed a CC-BY licence shall apply, and/or iii) in accordance with the terms applicable for US Federal Government officers or employees acting as part of their official duties; on a worldwide, perpetual, irrevocable, royalty-free basis to BMJ Publishing Group Ltd ("BMJ") its licensees and where the relevant Journal is co-owned by BMJ to the co-owners of the Journal, to publish the Work in this journal and any other BMJ products and to exploit all rights, as set out in our licence.

The Submitting Author accepts and understands that any supply made under these terms is made by BMJ to the Submitting Author unless you are acting as an employee on behalf of your employer or a postgraduate student of an affiliated institution which is paying any applicable article publishing charge ("APC") for Open Access articles. Where the Submitting Author wishes to make the Work available on an Open Access basis (and intends to pay the relevant APC), the terms of reuse of such Open Access shall be governed by a Creative Commons licence – details of these licences and which Creative Commons licence will apply to this Work are set out in our licence referred to above.

Other than as permitted in any relevant BMJ Author's Self Archiving Policies, I confirm this Work has not been accepted for publication elsewhere, is not being considered for publication elsewhere and does not duplicate material already published. I confirm all authors consent to publication of this Work and authorise the granting of this licence.

For peer review only

Title	Kobe Project for the Exploration of Newer Strategies to Reduce the Social Burden of Dementia: *cohort and intervention studies*
Authors'	Yoji Nagai (0000-0002-0836-0495), Shinsuke Kojima, Hisatomo Kowa (0000-0002-5300-8589), Yasuji Yamamoto, Hiroyuki Kajita (0000-0001-5470-4781), Tohmi Osaki, Yasumasa Kakei (0000-0002-5997-514X), Kavita U Kothari (0000-0002-0759-5225), Ryoma Kayano.
Address, Authors' names and positions	Clinical & Translational Research Center, Kobe University Hospital, 7-5-2 Kusunoki-cho, Chuo-ku, Kobe 650-0017, Japan Yoji Nagai Professor & Director,<br><br>Institute of Medical Research and Innovation, Translational Research Center for Medical Innovation, Foundation for Biomedical Research and Innovation, Kobe, Japan 1-5-4 Minatojima-minamimachi, Chuo-ku, Kobe, Hyogo, 650-0047 Japan Kojima Shinsuke Professor,<br><br>Division of Cognitive and Psychiatric Rehabilitation, Department of Rehabilitation Science, Kobe University Graduate School of Health Sciences Kowa Hisatomo Professor,<br><br>Department of Biosignal Pathophysiology, Graduate School of Medicine, Kobe University Hospital, 7-5-2 Kusunoki-cho, Chuo-ku, Kobe 650-0017, Japan Yamamoto Yasuji Professor,<br><br>Faculty of Rehabilitation, Kobe Gakuin University, 518 Arise, Ikawadani-cho, Nishi-ku, Kobe 651-2180 Japan Hiroyuki Kajita Assistant Professor,<br><br>Faculty of Rehabilitation, Kobe Gakuin University, 518 Arise, Ikawadani-cho, Nishi-ku, Kobe 651-2180 Japan Tohmi Osaki Assistant Professor,<br><br>Clinical & Translational Research Center, Kobe University Hospital, 7-5-2 Kusunoki-cho, Chuo-ku, Kobe 650-0017, Japan Yasumasa Kakei Assistant Professor,<br><br>Clinical & Translational Research Center, Kobe University Hospital, 7-5-2 Kusunoki-cho, Chuo-ku, Kobe 650-0017, Japan Kavita Kothari Technical Assistant,<br><br>World Health Organization Centre for Health Development (WHO Kobe Center), I.H.D. Centre Building 9F, 1-5-1 Wakinohama-Kaigandori, Chuo-ku, Kobe 651-0073 Japan Ryoma Kayano Technical officer.
Corresponding author	**Correspondence to:** Y Kakei, D.D.S., Ph.D, ykakei@med.kobe-u.ac.jp Clinical & Translational Research Center, Kobe University Hospital, 7-5-2 Kusunoki-cho, Chuo-ku, Kobe 650-0017, Japan. Tel: 078 - 382 6729

Short running title: Reducing the Burden of Dementia

For peer review only - http://bmjopen.bmj.com/site/about/guidelines.xhtml

Abstract

Introduction: This research project addresses the lack of screening tools for the early detection of high-risk individuals for long term care, through 4 individual studies.

Study 1 investigates the predictive ability of the "Kihon Check List", study 2 the "Cognitive Function instrument" & EQ-5D and study 3 the "Cognitive Function instrument" & EQ-5D as well as the "Frail Kenshin" health checkup, for incident long term care certification over a follow up period of up to 4 years. This is the first large prospective study to evaluate the predictive ability of these tools for the outcome measure long term care certification. The last subsection of this project study 4 aims to explore a mixed methods intervention for delaying the need for long term care. This section is purely exploratory looking for clues for further studies.

Methods and analysis: Baseline data have been collected through local government programs, as well as through postal self-reported questionnaires. The primary outcome variable for all studies is long-term care certification data. Statistical analysis will be carried out using Kaplan Meier, Multiple Cox regression as well as logistic regression.

Conclusion: This project hopes to identify tools effective in predicting long term care need. This will enable identification of citizens that are of higher risk for long term care in the near future. This subset of high-risk individuals can in the future be addressed for extra support/intervention.

Ethics & Dissemination: All studies have been approved by respective institutional ethical committees and the WHO ethical committee ERC.0002899. In addition, all studies conform to the provisions of the Declaration of Helsinki and are conducted in accordance with Japan's "Ethical Guidelines for Medical and Health Research Involving Human Subjects". All findings will be disseminated at conferences and published in peer-reviewed journals.

Key Words: dementia, elderly, long term care, population-based study, risk assessment.

For peer review only - http://bmjopen.bmj.com/site/about/guidelines.xhtml

Article Summary

"Strengths and Limitations of These Studies"

・To the best of our knowledge, this is the first large population-based study to assess the validity of several simple cognition related questionnaires for the prediction of long-term care.

・The various baseline measures give a broad instrument to use for the prediction of long-term care.

・As a low-cost rapid assessment study method was used, it will be possible to repeat the study at regular intervals to assess validity of the tools and identify high risk groups for early intervention.

・This project will contribute to better health by supporting early identification of high-risk groups for ageing societies in developed and low-income and middle-income countries.

・ The length of follow up of all the studies, which range from 2-4 years, is also a limiting factor. This project is further limited by study 4, since it is a small sample and limited single-center intervention study.

Word count: 3,137 words

For peer review only

Kobe Project for the Exploration of Newer Strategies to Reduce the Social Burden of Dementia: c*ohort and intervention studies*

Background and Purpose

Due to high life expectancies and low birth rates, Japan has a rapidly ageing population. Since 2010, the proportion of population 65 years and older increased over 21%, making Japan a super-aged society [1]. In 2019 this proportion reached 28% of the population [2]. One of the major health care challenges is an increased need for long term care (LTC).

For peer review only - http://bmjopen.bmj.com/site/about/guidelines.xhtml

Dementia is one of the leading causes for care-requiring status, accounting for nearly 17% of individuals in long-term care (LTC) [3]. Furthermore, the heart-breaking nature of the disease, accompanied with the significant impact on families and society, makes it a public health priority [4]. Japan has the highest dementia prevalence (2.3% of the population) among OECD countries in 2017, and is projected to reach 3.8% by 2037 [5].

In spite of the urgent need to reduce the social burden, there are currently no therapeutic strategies to halt or slow the disease process [6,7]. Lessons from prior studies recommend a focus on pre-clinical dementia and targeting of potential high-risk individuals who might benefit the most [8]. In line with such recommendations, the focus of dementia research is shifting to earlier stages of cognitive decline, in the hope of mitigating or even reversing the pathophysiological processes [9,10]. The achievements are still limited, in part due to the lack of efficient screening and evaluation methods for detecting higher risk individuals in society.

Under these circumstances, the Japanese Ministry of Health, Labor and Welfare (MHLW) issued a 5 year plan in 2013, for advancing measures against dementia, called "The Orange Plan" [11]. In accordance with this plan, every municipality began to provide a variety of civil services in an attempt to tackle dementia, hoping to reduce the increasing social burden. For example, Kobe, the 6th largest city in Japan, initiated social

For peer review only

For peer review only - http://bmjopen.bmj.com/site/about/guidelines.xhtml

participation programs, aiming to prevent or mitigate cognitive decline, including the "Kihon Check List" (KCL) program, brain health school program, and senior college program. The effects of such services, however, have not been adequately evaluated due to the lack of appropriate evaluation metrics, and thus, improvements were hampered. Furthermore, LTC need data, is independently certified in the municipal office, with no link to dementia or other health promotion civil services.

The current project was designed-to enable earlier identification of individuals at risk for dementia and consequently LTC. The first three studies use various cognition related questionnaires in an attempt to predict LTC need and identify individuals at higher risk. The last study is a small exploratory study which explores possible early interventions in these high-risk individuals to slow the progression of cognitive decline. This article introduces and outlines four individual studies included in this project.

For peer review only

Methods

The current project comprises of 4 individual studies focusing on cognition-related parameters and LTC need certification in healthy community dwelling citizens in Kobe city. Eligibility criteria of respective studies are independently defined, depending on the

For peer review only - http://bmjopen.bmj.com/site/about/guidelines.xhtml

purpose of each study. Studies 1 and 4a were approved by the Ethics Committee in February 2017 and Studies 2 and 3 in March 2017. All studies are currently ongoing.

Evaluation and Outcome Measures

Kihon Check List (KCL)

KCL is a self-reported frailty questionnaire designed by the MHLW, to identify frail elderly individuals at risk for LTC. It consists of a 25-item self-reported questionnaire, and has been widely delivered to citizens from local governments across the nation from 2006 - 2016. It covers several domains of instrumental activities for daily living (ADL), including general independence, physical strength, nutritional status, oral function, housebound, cognitive function, depression risk, and general health status. In particular, the KCL includes three cognitive function-related questions - Do your family or your friends point out your memory loss? Do you make a call by looking up phone numbers? Do you find yourself not knowing today's date?

Cognitive Function Instrument (CFI)

CFI is a screening tool to detect early changes of ADL due to cognitive decline [12]. It was developed by the Alzheimer's Disease Cooperative Study (ADCS) over the last decade. CFI consists of 14 questions that are related to functional ability changes as a

For peer review only - http://bmjopen.bmj.com/site/about/guidelines.xhtml

result of cognitive impairments. It is brief and easily administered at home through post, telephone or transmitted electronically. The CFI score is calculated by summing 1 point for “Yes”, 0 point for “No” and 0.5 for “Maybe.” Although the CFI questionnaire includes “self” and “partner” versions, this project uses the “self” version, because the current surveillance set up didn’t allow for both versions.

EQ-5D

EQ-5D is a standardized measure of health status developed by the EuroQol Group in order to provide a simple, generic measure of health for clinical and economic appraisal. It is applicable to a wide range of health conditions and treatments. EQ-5D is designed for self-completion and is suited for use in postal surveys, in clinics, and in face-to-face interviews. It is officially available in 171 languages and the official Japanese language version has been selected for the purpose of this study. The EuroQol Group provides two version of the EQ-5D with 3 or 5 dimensions, the latter of which (EQ-5D-5L) was adopted in this project. The EQ-5D-5L includes 5 categories with 5 questions in each category, summing up to 25 questions. Based on the pattern of answers, the health status of an individual is given a single index value, ranging from zero to one, death to perfect health.

Frail Kenshin

Frail Kenshin” is an elderly citizen health checkup program by Kobe city, which is

For peer review only - http://bmjopen.bmj.com/site/about/guidelines.xhtml

offered to citizens covered by the "Kokumin Kenko Hoken" insurance (Kokumin Kenko Hoken is the National Health Insurance system in Japan, it includes all Japanese citizens and long-term residents). It has physically accessed components as well as self-assessed questions related to frailty. It evaluates, motor function, nutrition, oral function, forgetfulness, mental health, chewing ability, dental health, swallowing ability, grip strength, height (cm), weight (kg), BMI and more.

Long-term Care Certification

Under the national LTC Insurance Act, certification of LTC need has been implemented since April 2000. The main beneficiaries are elderly individuals aged 65 or over. LTC need certification is routinely conducted by each municipal office on an application basis from citizens. LTC need status is categorized into 8 levels (no need for support or care: 0, support level: 1 or 2, care level: 1 to 5). In the current project, certified care levels from 1 (partial assistance) to 5 (total assistance with disabled communication) were defined as to be in need of LTC.

So far, identification of individuals at higher risk of dementia has been hampered, largely because of the lack of convenient screening methods. Appropriate evaluation measures play a crucial role for the success of this project. For example, measures for baseline evaluation need to be easy to use, easy to answer, and quantitative. Also, measures for

For peer review only - http://bmjopen.bmj.com/site/about/guidelines.xhtml

outcome evaluation need to be fitted for purpose, objective, and must be readily observable in local governmental services. As measures to meet such requirements, KCL, CFI, EQ-5D and LTC certification have been chosen as some of the baseline and/or outcome evaluation tools. KCL has been proven to be a reliable indicator of frailty [13], and is considered advantageous for a population-based approach [14]. EQ-5D is an established standard measure for health related QOL, and is widely used around the world. In contrast, CFI is a relatively new measure, which could be used in large epidemiological and clinical studies with minimal in-person contact. In a previous study by Amariglio et al [12], the CFI score was gradually worse in individuals who developed dementia than in those that did not, over a period of 4 years. Although their finding does not directly demonstrate the utility of CFI for risk assessment of dementia, it suggests value to be tested in larger prospective studies. However, it should be noted that CFI is a measure for the change of ADL due to cognitive decline, and not a direct measure for cognitive function per se. Thus, the results to be obtained need to be interpreted with caution, yet it is a potentially useful tool for the risk stratification of LTC need in the community.

For peer review only - http://bmjopen.bmj.com/site/about/guidelines.xhtml

Outline of Studies Included in the Project

The 4 studies are briefly explained below and summarized in Table 1.

Table1. Summary of studies

	Study 1	Study 2	Study 3	Study 4
**Target Population for recruitment**	General Population (80,000)	Citizens with Possible Cognitive Decline (4,131)	"Frail Check" Participants (4,800)	Brain health school program participants (80)
**Sample Population**	50,155	2,385	2,105	57
**Baseline Evaluation**	KCL	CFI, EQ-5D, GDS	CFI, EQ-5D, General Frailty Measures	MMSE, EQ-5D CFI, GDS, etc
**Primary Outcome**	LTC Need	LTC Need	LTC Need	MMSE
**Secondary Outcome**	LTC Need Level	CFI, EQ-5D	LTC Need Level	EQ-5D, CFI, Five Cog, etc
**Intervention**	None	None	None	Intensive Cognitive Training
**Study Period**	4-5 years	2 years	2 years	30 months

KCL: Kihon Check List, CFI: Cognitive Function Instrument, GDS: Geriatric Depression

For peer review only - http://bmjopen.bmj.com/site/about/guidelines.xhtml

Scale, MMSE: Mini Mental State Examination, LTC: Long-Term Care

Study 1: Elderly Cohort Study for Long Term Care Need Risk Stratification

This study is a prospective cohort study that investigates the predictive ability of the KCL and the cognitive domain therein for incident LTC certification. The target group was approximately 80,000 elderly Kobe citizens to whom KCL was sent in 2015.

Inclusion: The general population will be eligible for inclusion if they are aged 70 years or older, responded to the KCL of 2015, and were not LTC need certified at the time of the KCL data collection.

77,900 target population with an estimated response rate of 60%, thus 50,000 people are expected to participate in the study. The primary outcome is to be certified for LTC need. The secondary endpoints include levels (1 to 5) and causes (dementia or physical disability) of LTC need. The KCL data are collected via postal mail survey by the Kobe municipal office, which will be merged with LTC need certification data. The follow up duration is calculated from the date of first response to KCL to the date of LTC need certification until a cutoff date of Nov. 2019 (4 years).

For the statistical analysis, the incidence of LTC need at 1 ,2 ,3 and 4 years after KCL

For peer review only - http://bmjopen.bmj.com/site/about/guidelines.xhtml

distribution will be estimated using the Kaplan-Meier method. For the estimation, data for all eligible citizens will be included in the time-to-event analysis. The follow up duration is calculated from the date of first response to KCL to the date of LTC need certification up until a cutoff date of November 2019.

Subsequently, multivariate Cox regression analysis will be performed using the stepwise variable selection method to narrow down independent variables and to estimate the associated HR. Multivariate Cox Regression Analysis will be performed for 27 variables; age, sex and 25 questions of the KCL. Statistical analysis will be carried out using the SAS version 9.3 (SAS Institute Inc). The significance threshold is set at $P<0.05$.

Study 2: Quantifying the Risk of Long-Term Care Need in Citizens with Possible Cognitive Decline

This is a prospective cohort study which examines the predictive ability of CFI and EQ-5D questionnaires, for LTC certification, in elderly citizens at higher risk of dementia. The sample population is a subgroup population of those that responded to the KCL of 2015 (a subgroup of study 1). The target population are those that responded with unfavorable answers to either of the 3 cognitive domain questions in the KCL from the 2015 survey. The major inclusion criteria are citizens who are above or equal to 70 years old at the time of KCL survey, had at least one unfavorable answer to the

For peer review only

For peer review only - http://bmjopen.bmj.com/site/about/guidelines.xhtml

cognitive domain questions in the KCL, those that responded to CFI and EQ-5D questionnaires in 2017 and 2019, and are not LTC certified. The target population is 4,131 citizens which were sent CFI, and EQ-5D in 2017. Out of which 2,981 citizens responded. In 2019 this same cohort were sent the CFI and EQ-5D, out of which 2,444 citizens replied. After discounting for uncompleted forms, 2,292 formed the sample for this study. The primary endpoint is the occurrence of LTC need certification after 2 years, and the secondary endpoints are the change in CFI and EQ-5D during the same period. After matching for LTC certification, statistical analysis will be carried out with the use of proportional Hazard model. Relative risk for future LTC need and death is estimated in relation to the baseline CFI and EQ5D. The relationship between the changes in such parameters and LTC need will also explored. Repeated measure ANOVA or mixed effect model will be used to examine the changes of CFI and EQ5D, in relation to occurrence of LTC need.

Study 3: Quantifying the Risk of Long-Term Care Need in Generally Healthier Citizens

This study explores the relationship of CFI, EQ-5D and other frailty-related parameters, with the risk of future LTC need within 3 years. It will search for other prognostic factors that may predict the progression to LTC. The target population

For peer review only - http://bmjopen.bmj.com/site/about/guidelines.xhtml

was Kobe residents who turned 65 years old in 2017 or 20 18 and who underwent "Frail Kenshin". "Frail Kenshin" is an elderly citizen health checkup program by Kobe city, which is offered to citizens covered by the "Kokumin Kenko Hoken" insurance (Kokumin Kenko Hoken is the National Health Insurance system in Japan, it includes all Japanese citizens and long-term residents). "Kokumin Kenko Hoken" insurance covered citizens who turned 65 in 2017 or 2018 were 28,800 citizens, out of which 2,705 people appeared for the "Frail Kenshin" checkup of 2017 and 2018 and formed the sample for this study. Thus, the target citizens are younger than Study 1 and 2, and not known to be at risk for dementia. They are also likely to be health-conscious citizens since they appeared for a voluntary health check-up. The major inclusion criteria are those who turn or turned 65 years old during 2017 or 2018, underwent "Frail Kenshin" checkup and were not LTC certified at the time of the checkup. The primary endpoint is the occurrence of LTC need, and the secondary endpoints include LTC care levels (1 to 5). CFI and EQ-5D questionnaires were carried out at the venue of checkup, concomitant with other frailty checks, and the completed questionnaires were collected and checked by the person in charge.

First the incidence of LTC over the study period will be calculated using LTC care level 1 or greater. The statistical methods to be used are Logistic regression model

For peer review only - http://bmjopen.bmj.com/site/about/guidelines.xhtml

(estimated by LASSO) with the presence and absence of LTC care as the dependent variable with CFI Self score, EQ-5D scores, and other "Frail Kenshin" checkup results as explanatory variables. A formula and ROC curve will be estimated. Multiple regression will also be carried out for continuous variables used in the "Frail Kenshin" health checkup. In addition, age and gender are included in the model as explanatory variables in all analysis. In the case that the significance level of the above-mentioned test is P <0.05 on both sides, the multiplicity of the tests is not considered an issue.

SAS Version 9.4 and R version 3.6 are used for the analysis.

Study 4: Study on Effect of Multimodal Dementia Prevention Program in Community-Dwelling Elderly

This is a parallel exploratory intervention study, to explore the effects of multimodal interventions for slowing the process of cognitive decline. The target group were approximately 80 citizens over 70 years old who participated in a dementia prevention education program which was held monthly by Kobe city in 2016. Individuals who agreed to participate in this study are randomly divided into two groups. Both groups undergo an intensive training program (education, cognitive training and exercise) once a week for a total of 10 weeks and are monitored every 6 months for up to 30 months after recruitment. During the follow up period, the intervention group receives a booster

For peer review only - http://bmjopen.bmj.com/site/about/guidelines.xhtml

training once every 3 months, whereas the control group does not. The endpoints include changes in Mini Mental State Examination, CFI, EQ-5D, Five Cog, Timed Up & Go test, Physical Activity Scale for the Elderly and Geriatric Depression Scale. In the analysis of variance between the three groups, effect size was set at 0.25, α=0.05, and power=0.80, the necessary data number will be approximately 50 persons for each group. Since this is a long-term longitudinal study, the number of subjects is set at a higher 100 persons, which is about double the necessary number (for statistical significance) as a cautionary measure for drop outs and missing data. The longitudinal change of the evaluation results in each group is analyzed using repeated analysis of variance and mixed effects model. The amount of change in the evaluation result between groups is compared by multiple comparison tests: the paired t-test or Wilcoxon test.

Discussion

In a prior study (Kojima et al, 2019) an analysis of 182,099 Kobe citizens showed, cognitively unfavorable answers in KCL were found to be associated with the incidence of LTC need, independent of age, sex and other items on KCL [15]. Furthermore, as the number of unfavorable answers to the cognitive domain increased from 0 to 1, 2 and 3, the incidence of LTC need progressively increased from 3.5% to 6.4%, 12.6%, and 29.6% after 3 years. This finding suggests a potential utility for KCL in the risk stratification

For peer review only - http://bmjopen.bmj.com/site/about/guidelines.xhtml

of future LTC need. However, because this was a retrospective analysis on pooled data, the causal relationship between cognitively unfavorable answers and LTC need could not be validated. The current project will test the predictive ability of the KCL as well as other tools such as CFI, EQ-5D and "Frail Kenshin" checkup in relation to LTC need.

Each of the 4 studies in this project focuses on citizens at a different level of dementia risk (Study 1: general population, Study 2: higher risk population, Study 3: lower risk population, Study 4: higher risk individuals willing to participate in the training program), see Table 1.

Strengths & weakness of the study

A limitation of the study is that the follow up study period is relatively short, due to the contract duration by the funder. However, if this evaluation system is incorporated into local governmental systems in the future, follow up periods can be virtually unlimited, and baseline and outcome evaluation would be repeated (Figure 1). Through this process a quantitative feedback mechanism could be created forming the foundation to reduce the social burden of dementia, which further on, could also be extended to address other public health problem areas [16,17]. Additionally, Study 4 is a highly exploratory study with limited sample size. This study hopes to find what is feasible and possibly provide clues for improving the social burden of LTC need, which can then be tested in future

For peer review only - http://bmjopen.bmj.com/site/about/guidelines.xhtml

studies.

Meaning of study

By the completion of Study 1, 2 and 3, risk stratification of earlier cognitive decline for future LTC need will be improved on a population basis. Particularly, if individuals at risk for future dementia could be extracted, social efforts could be directed to such individuals who may receive a greater benefit, allowing for a more efficient allocation of limited public resources. Also, given the failure of many clinical trials for dementia drug development, the system constructed by this project could help prepare “trial-ready cohorts”, to increase efficiency of recruiting eligible participants.

In summary, this project hopes to show a risk prediction and early intervention model linked to the outcome measurement, LTC. If continuously implemented, it can be a foundation for addressing the burden of dementia earlier. The final hope of this project is “not to be demented”, but if demented, “not to be in need of care”.

For peer review only

References

1 Nishimura S. Social Security In Japan. Published Online First: March 2011.http://www.ipss.go.jp/s-info/e/Jasos2011/ss2011.pdf (accessed 31 Dec 2014).

2 The World Bank. Population ages 65 and above, total. World Bank Data. 2018.https://data.worldbank.org/indicator/SP.POP.65UP.TO.ZS?end=2019&locations=JP&start=2019 (accessed 22 Feb 2021).

3 Cabinet Office. Health and Welfare of the Elderly | The White Paper on the Aged Society in 2016 (The whole edition). 2020. http://www8.cao.go.jp/kourei/whitepaper/w-2016/html/zenbun/s1_2_3.html (accessed 22 Feb 2021).

4 World Health Organization - Alzheimer's Disease International. DEMENTIA: A Public Health Priority. Published Online First: 2012.http://apps.who.int/iris/bitstream/10665/75263/1/9789241564458_eng.pdf?ua=1 (accessed 6 Feb 2017).

5 Fukawa T. Prevalence of dementia among the elderly population in Japan. *Heal Prim Care* 2018;**2**. doi:10.15761/hpc.1000147

6 Rubinstein E, Duggan C, Landingham B Van, *et al.* A CALL TO ACTION THE GLOBAL RESPONSE TO DEMENTIA THROUGH POLICY INNOVATION. http://www.mhinnovation.net/sites/default/files/downloads/resource/WISH_Dementia_Forum_Report_08.01.15_WEB.pdf (accessed 3 Apr 2017).

7 UK Government. G8 dementia summit: Global action against dementia - 11 December 2013. Published Online First: 2013.https://www.gov.uk/government/publications/g8-dementia-summit-global-action-against-dementia/g8-dementia-summit-global-action-against-dementia-11-december-2013 (accessed 6 Feb 2017).

8 Imtiaz B, Tolppanen A-M, Kivipelto M, *et al.* Future directions in Alzheimer's disease from risk factors to prevention. *Biochem Pharmacol* 2014;**88**:661–70. doi:10.1016/j.bcp.2014.01.003

9 Urakami K. [Prevention of dementia]. *Nihon Rinsho* 2016;**74**:395–8.http://www.ncbi.nlm.nih.gov/pubmed/27025075 (accessed 2 Feb 2017).

10 Wu Y-T, Fratiglioni L, Matthews FE, *et al.* Dementia in western Europe:

For peer review only - http://bmjopen.bmj.com/site/about/guidelines.xhtml

epidemiological evidence and implications for policy making. *Lancet Neurol* 2016;**15**:116–24. doi:10.1016/S1474-4422(15)00092-7

11 Nakanishi M, Nakashima T. Features of the Japanese national dementia strategy in comparison with international dementia policies: How should a national dementia policy interact with the public health- and social-care systems? *Alzheimer's Dement* 2014;**10**:468-476.e3. doi:10.1016/j.jalz.2013.06.005

12 Amariglio RE, Donohue MC, Marshall GA, *et al.* Tracking early decline in cognitive function in older individuals at risk for Alzheimer disease dementia: the Alzheimer's Disease Cooperative Study Cognitive Function Instrument. *JAMA Neurol* 2015;**72**:446–54. doi:10.1001/jamaneurol.2014.3375

13 Sampaio PYS, Sampaio RAC, Yamada M, *et al.* Systematic review of the Kihon Checklist: Is it a reliable assessment of frailty? *Geriatr Gerontol Int* 2016;**16**:893–902. doi:10.1111/ggi.12833

14 Satake S, Shimokata H, Senda K, *et al.* Validity of Total Kihon Checklist Score for Predicting the Incidence of 3-Year Dependency and Mortality in a Community-Dwelling Older Population. *J Am Med Dir Assoc* 2017;**18**:552.e1-552.e6. doi:10.1016/j.jamda.2017.03.013

15 Kojima S, Murotani K, Zhou B, *et al.* Assessing long-term care risk in older individuals with possible cognitive decline: A large population-based study using the Kihon Checklist. *Geriatr Gerontol Int* 2019;**19**. doi:10.1111/ggi.13677

16 Msanori Fukushima, Kenichi Kohno, Shinsuke Kojima, Keisuke Yuasa, Kenichiro Tanabe YN. For the sharp decrease in the social burden of medical expenses, nursing care - Design of the area data utilization hubs and the infrastructure development to provide curative treatment. *Clin Eval* 2019;**47**:105–15.http://cont.o.oo7.jp/47sup36/47sup36contents_e.html (accessed 8 Jul 2020).

17 Fukushima. M. The reality of 2040 issues in Japan - Building the learning health society as a solution. *Clin Eval (in Press* 2020.

Author Statement

YK, YN, SK, HK, YY, HK, TO & RK contributed to the conception and design of this

For peer review only - http://bmjopen.bmj.com/site/about/guidelines.xhtml

research project and the four individual studies therein. YN, YK and KK participated in the preparation of the manuscript, and all authors critically revised the manuscript for important intellectual content. YK is the corresponding author and YK and YN are the guarantors of the work. The corresponding author attests that all listed authors meet authorship criteria and that no others meeting the criteria have been omitted.

Acknowledgements

We express our sincere gratitude to Miyuki Kawabata for the overall project management, and Ayumi Okada for the data management of respective studies in this project. Also, we are deeply grateful for the robust corporation of Kobe city municipal office, including the data collection, processing and anonymization, as well as all other efforts for the planning and implementation of this project.

Funding

This work was funded by the_World Health Organization and is supported by the World Health Organization Centre for Health Development (WHO Kobe Centre) grant number WKC: K18001. ERC.0002899. The Sponsor provided funding, but had no role in the research project design, or preparation and submission of the manuscript. The researchers are independent from the funders and all authors, internal and external, had full access to all the data in the study and can take responsibility for the integrity of the data and the accuracy of the data analysis.

Conflicts of Interest

"All authors have completed the ICMJE uniform disclosure form at www.icmje.org/coi_disclosure.pdf and declare: no support from any organisation for the submitted work; no financial relationships with any organisations that might have an interest in the submitted work in the previous three years; no other relationships or activities that could appear to have influenced the submitted work."

Transparency declaration

Dr. Yoji Nagai affirms that the manuscript is honest, accurate, and a transparetns account of the study being reported; no important aspects of the study has been omitted; any discrepancies from the study as originally planned have been explained.

For peer review only - http://bmjopen.bmj.com/site/about/guidelines.xhtml

Copyright/liscense for publication

The Corresponding Author has the right to grant on behalf of all authors and does grant on behalf of all authors, a worldwide licence to the Publishers and its licensees in perpetuity, in all forms, formats and media (whether known now or created in the future), to i) publish, reproduce, distribute, display and store the Contribution, ii) translate the Contribution into other languages, create adaptations, reprints, include within collections and create summaries, extracts and/or, abstracts of the Contribution, iii) create any other derivative work(s) based on the Contribution, iv) to exploit all subsidiary rights in the Contribution, v) the inclusion of electronic links from the Contribution to third party material where-ever it may be located; and, vi) licence any third party to do any or all of the above.

Figure & Table Legend:

Table1: Summary of studies

Figure 1. Project Concept. This figure depicts the concept of this project. If baseline and outcome evaluation can we repeated and incorporated into government practice, a foundation for the focus on high risk individuals and possible reduction in LTC (social burden) can be realized.

For peer review only

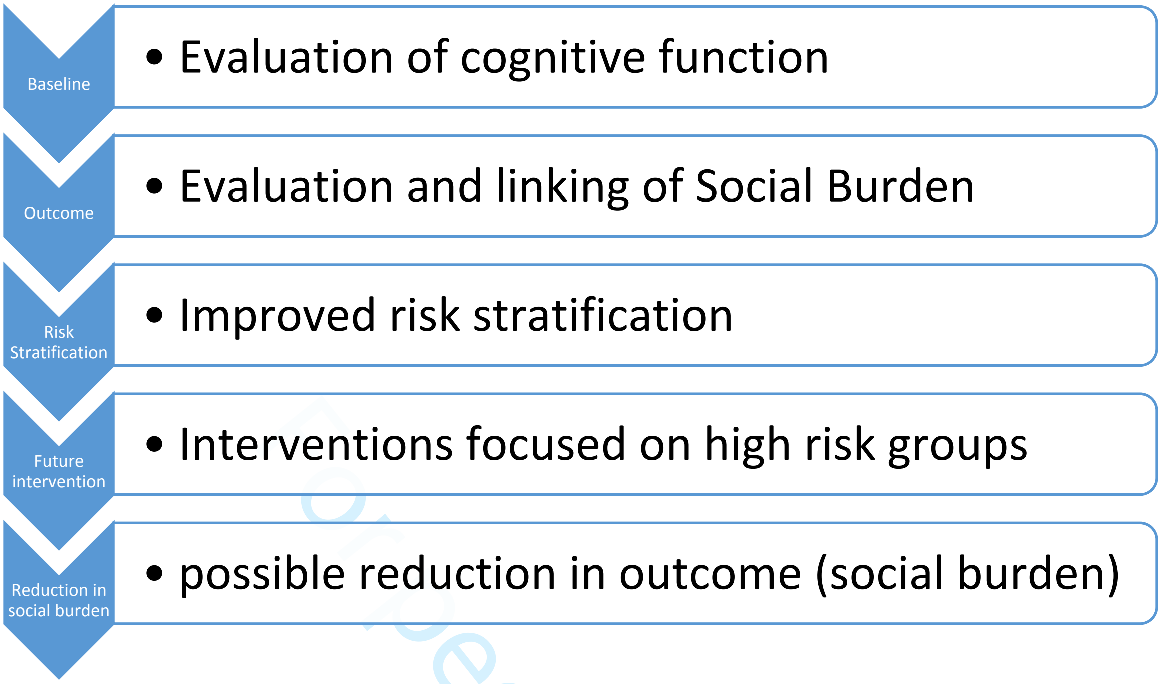

Figure 1. Project Concept. This figure depicts the concept of this project. If baseline and outcome evaluation can we repeated and incorporated into government practice, a foundation for the focus on high risk individuals and possible reduction in LTC (social burden) can be realized.

For peer review only

BMJ Open

Kobe Project for the Exploration of Newer Strategies to Reduce the Social Burden of Dementia: A study protocol of cohort and intervention studies.

Journal:	*BMJ Open*
Manuscript ID	bmjopen-2021-050948.R1
Article Type:	Protocol
Date Submitted by the Author:	18-May-2021
Complete List of Authors:	Nagai, Yoji; Kobe University Hospital, Clinical & Translational Research Center<br>Kojima, Shunsuke; Translational Research Center for Medical Innovation, Foundation for Biomedical Research and Innovation, Institute of Medical Research and Innovation<br>Kowa, Hisatomo; Kobe Univerity Graduate School of Health Sciences, Division of Cognitive and Psychiatric Rehabilitation, Department of Rehabilitation Science<br>Yamamoto, Yasuji; Kobe University Graduate School of Medicine School of Medicine, Department of Biosignal Pathophysiology<br>Kajita, Hiroyuki ; Kobe Gakuin University, Faculty of Rehabilitation<br>Osaki, Tohmi; Kobe Gakuin University Faculty of Rehabilitation Graduate School of Rehabilitation, Faculty of Rehabilitation<br>Kakei, Yasumasa; Kobe University Hospital, Clinical & Translational Research Center<br>Kothari, Kavita; Kobe University Hospital, Clinical & Translational Research Center<br>Kayano, Ryoma ; WHO Kobe Center
**Primary Subject Heading**:	Epidemiology
Secondary Subject Heading:	Public health, Mental health
Keywords:	Dementia < NEUROLOGY, EPIDEMIOLOGY, Neuropathology < NEUROLOGY, Health policy < HEALTH SERVICES ADMINISTRATION & MANAGEMENT, Protocols & guidelines < HEALTH SERVICES ADMINISTRATION & MANAGEMENT

SCHOLARONE™
Manuscripts

I, the Submitting Author has the right to grant and does grant on behalf of all authors of the Work (as defined in the below author licence), an exclusive licence and/or a non-exclusive licence for contributions from authors who are: i) UK Crown employees; ii) where BMJ has agreed a CC-BY licence shall apply, and/or iii) in accordance with the terms applicable for US Federal Government officers or employees acting as part of their official duties; on a worldwide, perpetual, irrevocable, royalty-free basis to BMJ Publishing Group Ltd ("BMJ") its licensees and where the relevant Journal is co-owned by BMJ to the co-owners of the Journal, to publish the Work in this journal and any other BMJ products and to exploit all rights, as set out in our licence.

The Submitting Author accepts and understands that any supply made under these terms is made by BMJ to the Submitting Author unless you are acting as an employee on behalf of your employer or a postgraduate student of an affiliated institution which is paying any applicable article publishing charge ("APC") for Open Access articles. Where the Submitting Author wishes to make the Work available on an Open Access basis (and intends to pay the relevant APC), the terms of reuse of such Open Access shall be governed by a Creative Commons licence – details of these licences and which Creative Commons licence will apply to this Work are set out in our licence referred to above.

Other than as permitted in any relevant BMJ Author's Self Archiving Policies, I confirm this Work has not been accepted for publication elsewhere, is not being considered for publication elsewhere and does not duplicate material already published. I confirm all authors consent to publication of this Work and authorise the granting of this licence.

For peer review only

Title

Kobe Project for the Exploration of Newer Strategies to Reduce the Social Burden of Dementia: *A study protocol of cohort and intervention studies.*

Authors'

Yoji Nagai (0000-0002-0836-0495), Shinsuke Kojima, Hisatomo Kowa (0000-0002-5300-8589), Yasuji Yamamoto, Hiroyuki Kajita (0000-0001-5470-4781), Tohmi Osaki, Yasumasa Kakei (0000-0002-5997-514X), Kavita U Kothari (0000-0002-0759-5225), Ryoma Kayano.

Address, Authors' names and positions

Clinical & Translational Research Center, Kobe University Hospital, 7-5-2 Kusunoki-cho, Chuo-ku, Kobe 650-0017, Japan Yoji Nagai Professor & Director,

Institute of Medical Research and Innovation, Translational Research Center for Medical Innovation, Foundation for Biomedical Research and Innovation, Kobe, Japan 1-5-4 Minatojima-minamimachi, Chuo-ku, Kobe, Hyogo, 650-0047 Japan Kojima Shinsuke Professor,

Division of Cognitive and Psychiatric Rehabilitation, Department of Rehabilitation Science, Kobe University Graduate School of Health Sciences Kowa Hisatomo Professor,

Department of Biosignal Pathophysiology, Graduate School of Medicine, Kobe University Hospital, 7-5-2 Kusunoki-cho, Chuo-ku, Kobe 650-0017, Japan Yamamoto Yasuji Professor,

Faculty of Rehabilitation, Kobe Gakuin University, 518 Arise, Ikawadani-cho, Nishi-ku, Kobe 651-2180 Japan Hiroyuki Kajita Assistant Professor,

Faculty of Rehabilitation, Kobe Gakuin University, 518 Arise, Ikawadani-cho, Nishi-ku, Kobe 651-2180 Japan Tohmi Osaki Assistant Professor,

Clinical & Translational Research Center, Kobe University Hospital, 7-5-2 Kusunoki-cho, Chuo-ku, Kobe 650-0017, Japan Yasumasa Kakei Assistant Professor,

Clinical & Translational Research Center, Kobe University Hospital, 7-5-2 Kusunoki-cho, Chuo-ku, Kobe 650-0017, Japan Kavita Kothari Technical Assistant,

World Health Organization Centre for Health Development (WHO Kobe Center), I.H.D. Centre Building 9F, 1-5-1 Wakinohama-Kaigandori, Chuo-ku, Kobe 651-0073 Japan Ryoma Kayano Technical officer.

Corresponding author

Correspondence to: Y Kakei, D.D.S., Ph.D, ykakei@med.kobe-u.ac.jp
Clinical & Translational Research Center, Kobe University Hospital, 7-5-2 Kusunoki-cho, Chuo-ku, Kobe 650-0017, Japan. Tel: 078 - 382 6729

Short running title: Reducing the Burden of Dementia

For peer review only

For peer review only - http://bmjopen.bmj.com/site/about/guidelines.xhtml

Abstract

Introduction: This research project addresses the lack of screening tools for the early detection of high-risk individuals for long term care, through 4 individual studies.

Study 1 investigates the predictive ability of the "Kihon Check List", study 2 the "Cognitive Function instrument" & EQ-5D and study 3 the "Cognitive Function instrument" & EQ-5D as well as the "Frail Kenshin" health checkup, for incident long term care certification over a follow up period of up to 4 years. This is the first large prospective study to evaluate the predictive ability of these tools for the outcome measure long term care certification. The last subsection of this project study 4 aims to explore a mixed methods intervention for delaying the need for long term care. This section is purely exploratory, looking for clues for further studies.

Methods and analysis: Baseline data have been collected through local government programs, as well as through postal self-reported questionnaires. The primary outcome variable for all studies is long-term care certification data. Statistical analysis will be carried out using Kaplan Meier, Multiple Cox regression as well as logistic regression.

Conclusion: This project hopes to identify tools effective in predicting long term care need. This will enable identification of citizens that are of higher risk for long term care in the near future. This subset of high-risk individuals can in the future be addressed for extra support/intervention.

Ethics & Dissemination: All studies have been approved by respective institutional ethical committees and the WHO ethical committee ERC.0002899. In addition, all studies conform to the provisions of the Declaration of Helsinki and are conducted in accordance with Japan's "Ethical Guidelines for Medical and Health Research Involving Human Subjects". All findings will be disseminated at conferences and published in peer-reviewed journals.

Key Words: dementia, elderly, long term care, population-based study, risk assessment.

Study 4 Trial registration number: UMIN000023283

For peer review only - http://bmjopen.bmj.com/site/about/guidelines.xhtml

Article Summary

"Strengths and Limitations of These Studies"

- This research project includes population based studies that allow for simple and reliable assessments of the risk for future long-term care need.
- This research project utilizes big data which are collected by the local government (municipal office).
- The various baseline measures give a broad instrument to use for the prediction of long-term care needs concomitant with cognitive decline.
- The duration of follow-up of respective studies, which range from 2-4 years, is limited and further longer term studies will be required.
- Study 4 has limitations, since it is a small sample and limited single-center intervention study.

For peer review only

Word count: 3,192 words

For peer review only - http://bmjopen.bmj.com/site/about/guidelines.xhtml

Kobe Project for the Exploration of Newer Strategies to Reduce the Social Burden of Dementia: *A study protocol of cohort and intervention studies.*

Background and Purpose

Due to high life expectancies and low birth rates, Japan has a rapidly ageing population. Since 2010, the proportion of population 65 years and older increased over 21%, making Japan a super-aged society [1]. In 2019 this proportion reached 28% of the population [2]. One of the major health care challenges is an increased need for long term care (LTC). Dementia is one of the leading causes for care-requiring status, accounting for nearly 17% of individuals in long-term care (LTC) [3]. Furthermore, the heart-breaking nature of the disease, accompanied with the significant impact on families and society, makes it a public health priority [4]. Japan has the highest dementia prevalence (2.3% of the population) among OECD countries in 2017, and is projected to reach 3.8% by 2037 [5].

In spite of the urgent need to reduce the social burden, there are currently no therapeutic strategies to halt or slow the disease process [6,7]. Lessons from prior studies recommend a focus on pre-clinical dementia and targeting of potential high-risk individuals who might benefit the most [8]. In line with such recommendations, the focus of dementia research is shifting to earlier stages of cognitive decline, in the hope of mitigating or even reversing the pathophysiological processes [9,10]. The achievements are still limited, in part due to the lack of efficient screening and evaluation methods for detecting higher risk individuals in

For peer review only - http://bmjopen.bmj.com/site/about/guidelines.xhtml

society.

Under these circumstances, the Japanese Ministry of Health, Labor and Welfare (MHLW) issued a 5 year plan in 2013, for advancing measures against dementia, called "The Orange Plan" [11]. In accordance with this plan, every municipality began to provide a variety of civil services in an attempt to tackle dementia, hoping to reduce the increasing social burden. For example, Kobe, the 6th largest city in Japan, initiated social participation programs, aiming to prevent or mitigate cognitive decline, including the “Kihon Check List” (KCL) program, brain health school program, and senior college program. The effects of such services, however, have not been adequately evaluated due to the lack of appropriate evaluation metrics, and thus, improvements were hampered. Furthermore, LTC need data, is independently certified in the municipal office, with no link to dementia or other health promotion civil services.

The current project was designed to enable earlier identification of individuals at risk for dementia and consequently LTC. The first three studies use various cognition related questionnaires in an attempt to predict LTC need and identify individuals at higher risk. The last study is a small exploratory study which explores possible early interventions in these high-risk individuals to slow the progression of cognitive decline. This article introduces and outlines four individual studies included in this project.

For peer review only - http://bmjopen.bmj.com/site/about/guidelines.xhtml

Methods

The current project comprises of 4 individual studies focusing on cognition-related parameters and LTC need certification in healthy community dwelling citizens in Kobe city. Eligibility criteria of respective studies are independently defined, depending on the purpose of each study. Studies 1 and 4a were approved by the Ethics Committee in February 2017 and Studies 2 and 3 in March 2017. All studies are currently ongoing.

Evaluation and Outcome Measures

(Questionnaires used in these studies are available in the online supplementary file)

Kihon Check List (KCL)

KCL is a self-reported frailty questionnaire designed by the MHLW, to identify frail elderly individuals at risk for LTC. The questionnaire used can be found in *Supplementary file 1*. It consists of a 25-item self-reported questionnaire and has been widely delivered to citizens from local governments across the nation from 2006 - 2016. It covers several domains of instrumental activities for daily living (ADL), including general independence, physical strength, nutritional status, oral function, housebound, cognitive function, depression risk, and general health status. In particular, the KCL includes three cognitive function-related questions - Do your family or your friends point out your memory loss? Do you make a call

For peer review only - http://bmjopen.bmj.com/site/about/guidelines.xhtml

by looking up phone numbers? Do you find yourself not knowing today's date?

Cognitive Function Instrument (CFI)

CFI is a screening tool to detect early changes of ADL due to cognitive decline [12]. It was developed by the Alzheimer's disease Cooperative Study (ADCS) over the last decade. The questionnaire used can be found in *Supplementary file 2.* CFI consists of 14 questions that are related to functional ability changes as a result of cognitive impairments. It is brief and easily administered at home through post, telephone or transmitted electronically. The CFI score is calculated by summing 1 point for "Yes", 0 point for "No" and 0.5 for "Maybe." Although the CFI questionnaire includes "self" and "partner" versions, this project uses the "self" version, because the current surveillance set up didn't allow for both versions.

EQ-5D

EQ-5D is a standardized measure of health status developed by the EuroQol Group in order to provide a simple, generic measure of health for clinical and economic appraisal. It is applicable to a wide range of health conditions and treatments. The questionnaire used can be found in *Supplementary file 3.* EQ-5D is designed for self-completion and is suited for use in postal surveys, in clinics, and in face-to-face interviews. It is officially available in 171 languages and the official Japanese language version has been selected for the purpose of

For peer review only - http://bmjopen.bmj.com/site/about/guidelines.xhtml

this study. The EuroQol Group provides two version of the EQ-5D with 3 or 5 dimensions, the latter of which (EQ-5D-5L) was adopted in this project. The EQ-5D-5L includes 5 categories with 5 questions in each category, summing up to 25 questions. Based on the pattern of answers, the health status of an individual is given a single index value, ranging from zero to one, death to perfect health.

Frail *Kenshin*

The "Frail Kenshin" checkup is an elderly citizen health checkup program by Kobe city, which is offered to citizens covered by the "Kokumin Kenko Hoken" insurance (Kokumin Kenko Hoken is the National Health Insurance system in Japan, it includes all Japanese citizens and long-term residents). The questionnaires used along with this check up can be found in *Supplementary file 4 & 5.* It has physically accessed components as well as self-assessed questions related to frailty. It evaluates, motor function, nutrition, oral function, forgetfulness, mental health, chewing ability, dental health, swallowing ability, grip strength, height (cm), weight (kg), BMI and more.

Long-term Care Certification

Under the national LTC Insurance Act, certification of LTC need has been implemented since April 2000. The main beneficiaries are elderly individuals aged 65 or over. LTC need certification is routinely conducted by each municipal office on an application basis from

For peer review only - http://bmjopen.bmj.com/site/about/guidelines.xhtml

citizens. LTC need status is categorized into 8 levels (no need for support or care: 0, support level: 1 or 2, care level: 1 to 5). In the current project, certified care levels from 1 (partial assistance) to 5 (total assistance with disabled communication) were defined as to be in need of LTC.

So far, identification of individuals at higher risk of dementia has been hampered, largely because of the lack of convenient screening methods. Appropriate evaluation measures play a crucial role for the success of this project. For example, measures for baseline evaluation need to be easy to use, easy to answer, and quantitative. Also, measures for outcome evaluation need to be fitted for purpose, objective, and must be readily observable in local governmental services. As measures to meet such requirements, KCL, CFI, EQ-5D and LTC certification have been chosen as some of the baseline and/or outcome evaluation tools. KCL has been proven to be a reliable indicator of frailty [13], and is considered advantageous for a population-based approach [14]. EQ-5D is an established standard measure for health related QOL, and is widely used around the world. In contrast, CFI is a relatively new measure, which could be used in large epidemiological and clinical studies with minimal in-person contact. In a previous study by Amariglio et al [12], the CFI score was gradually worse in individuals who developed dementia than in those that did not, over a period of 4 years. Although their finding does not directly demonstrate the utility of CFI for risk assessment of

For peer review only - http://bmjopen.bmj.com/site/about/guidelines.xhtml

dementia, it suggests value to be tested in larger prospective studies. However, it should be noted that CFI is a measure for the change of ADL due to cognitive decline, and not a direct measure for cognitive function per se. Thus, the results to be obtained need to be interpreted with caution, yet it is a potentially useful tool for the risk stratification of LTC need in the community.

Outline of Studies Included in the Project

The 4 studies are briefly explained below and summarized in Table 1.

Table 1: Summary of studies

	Study 1	Study 2	Study 3	Study 4
**Target Population for recruitment**	General Population (80,000)	Citizens with Possible Cognitive Decline (4,131)	"Frail Check" Participants (4,800)	Brain health school program participants (80)
**Sample population**	50,155	2,385	2,105	57
**Baseline Evaluation**	KCL	CFI, EQ-5D, GDS	CFI, EQ-5D, General Frailty Measures	MMSE, EQ-5D CFI, GDS, etc
**Primary Outcome**	LTC Need	LTC Need	LTC Need	MMSE
**Secondary Outcome**	LTC Need Level	CFI, EQ-5D	LTC Need Level	EQ-5D, CFI, Five Cog, etc
**Intervention**	None	None	None	Intensive Cognitive Training
**Study Period**	4-5 years	2 years	2 years	30 months

For peer review only - http://bmjopen.bmj.com/site/about/guidelines.xhtml

KCL: Kihon Check List, CFI: Cognitive Function Instrument, GDS: Geriatric Depression Scale, MMSE: Mini Mental State Examination, LTC: Long-Term Care

Study 1: Elderly Cohort Study for Long Term Care Need Risk Stratification

This study is a prospective cohort study that investigates the predictive ability of the KCL and the cognitive domain therein for incident LTC certification. The target group was approximately 80,000 elderly Kobe citizens to whom KCL was sent in 2015.

Inclusion: The general population will be eligible for inclusion if they are aged 70 years or older, responded to the KCL of 2015, and were not LTC need certified at the time of the KCL data collection.

77,900 target population with an estimated response rate of 60%, thus 50,000 people are expected to participate in the study. The primary outcome is to be certified for LTC need. The secondary endpoints include levels (1 to 5) and causes (dementia or physical disability) of LTC need. The KCL data are collected via postal mail survey by the Kobe municipal office, which will be merged with LTC need certification data. The follow up duration is calculated from the date of first response to KCL to the date of LTC need certification until a cutoff date of Nov. 2019 (4 years).

For the statistical analysis, the incidence of LTC need at 1, 2, 3 and 4 years after KCL

For peer review only - http://bmjopen.bmj.com/site/about/guidelines.xhtml

distribution will be estimated using the Kaplan-Meier method. For the estimation, data for all eligible citizens will be included in the time-to-event analysis. The follow up duration is calculated from the date of first response to KCL to the date of LTC need certification up until a cutoff date of November 2019.

Subsequently, multivariate Cox regression analysis will be performed using the stepwise variable selection method to narrow down independent variables and to estimate the associated HR. Multivariate Cox Regression Analysis will be performed for 27 variables; age, sex and 25 questions of the KCL. Statistical analysis will be carried out using the SAS version 9.3 (SAS Institute Inc). The significance threshold is set at $P<0.05$.

Study 2: Quantifying the Risk of Long-Term Care Need in Citizens with Possible Cognitive Decline

This is a prospective cohort study which examines the predictive ability of CFI and EQ-5D questionnaires, for LTC certification, in elderly citizens at higher risk of dementia. The sample population is a subgroup population of those that responded to the KCL of 2015 (a subgroup of study 1). The target population are those that responded with unfavorable answers to either of the 3 cognitive domain questions in the KCL from the 2015 survey. The major inclusion criteria are citizens who are above or equal to 70 years old at the time of KCL survey, had at least one unfavorable answer to the cognitive domain questions in the

For peer review only - http://bmjopen.bmj.com/site/about/guidelines.xhtml

KCL, those that responded to CFI and EQ-5D questionnaires in 2017 and 2019, and are not LTC certified. The target population is 4,131 citizens which were sent CFI, and EQ-5D in 2017. Out of which 2,981 citizens responded. In 2019 this same cohort were sent the CFI and EQ-5D, out of which 2,444 citizens replied. After discounting for uncompleted forms, 2,292 formed the sample for this study. The primary endpoint is the occurrence of LTC need certification after 2 years, and the secondary endpoints are the change in CFI and EQ-5D during the same period. After matching for LTC certification, statistical analysis will be carried out with the use of proportional Hazard model. Relative risk for future LTC need and death is estimated in relation to the baseline CFI and EQ5D. The relationship between the changes in such parameters and LTC need will also explored. Repeated measure ANOVA or mixed effect model will be used to examine the changes of CFI and EQ5D, in relation to occurrence of LTC need.

Study 3: Quantifying the Risk of Long-Term Care Need in Generally Healthier Citizens

This study explores the relationship of CFI, EQ-5D and other frailty-related parameters, with the risk of future LTC need within 3 years. It will search for other prognostic factors that may predict the progression to LTC.   The target population was Kobe residents who turned 65 years old in 2017 or 20 18 and who underwent “Frail Kenshin”. “Frail Kenshin” is an elderly citizen health checkup program by Kobe city, which is offered to citizens

For peer review only

For peer review only - http://bmjopen.bmj.com/site/about/guidelines.xhtml

covered by the “Kokumin Kenko Hoken” insurance (Kokumin Kenko Hoken is the National Health Insurance system in Japan, it includes all Japanese citizens and long-term residents). "Kokumin Kenko Hoken" insurance covered citizens who turned 65 in 2017 or 2018 were 28,800 citizens, out of which 2,705 people appeared for the "Frail Kenshin" checkup of 2017 and 2018 and formed the sample for this study. Thus, the target citizens are younger than Study 1 and 2, and not known to be at risk for dementia. They are also likely to be health-conscious citizens since they appeared for a voluntary health check-up. The major inclusion criteria are those who turn or turned 65 years old during 2017 or 2018, underwent “Frail Kenshin” checkup and were not LTC certified at the time of the checkup. The primary endpoint is the occurrence of LTC need, and the secondary endpoints include LTC care levels (1 to 5). CFI and EQ-5D questionnaires were carried out at the venue of checkup, concomitant with other frailty checks, and the completed questionnaires were collected and checked by the person in charge.

First the incidence of LTC over the study period will be calculated using LTC care level 1 or greater. The statistical methods to be used are Logistic regression model (estimated by LASSO) with the presence and absence of LTC care as the dependent variable with CFI Self score, EQ-5D scores, and other “Frail Kenshin” checkup results as explanatory variables. A formula and ROC curve will be estimated. Multiple regression will also be

For peer review only - http://bmjopen.bmj.com/site/about/guidelines.xhtml

carried out for continuous variables used in the "Frail Kenshin" health checkup. In addition, age and gender are included in the model as explanatory variables in all analysis. In the case that the significance level of the above-mentioned test is P <0.05 on both sides, the multiplicity of the tests is not considered an issue. SAS Version 9.4 and R version 3.6 are used for the analysis.

Study 4: Study on Effect of Multimodal Dementia Prevention Program in Community-Dwelling Elderly

This study is registered as a clincal trial on the UMIN Clinical Trial Registry: UMIN000023283, with Receipt No.: R000026827.

This is a parallel exploratory intervention study, to explore the effects of multimodal interventions for slowing the process of cognitive decline. The target group were approximately 80 citizens over 70 years old who participated in a dementia prevention education program which was held monthly by Kobe city in 2016. Individuals who agreed to participate in this study are randomly divided into two groups. Both groups undergo an intensive training program (education, cognitive training and exercise) once a week for a total of 10 weeks and are monitored every 6 months for up to 30 months after recruitment. During the follow up period, the intervention group receives a booster training once every 3 months, whereas the control group does not. The endpoints include changes in Mini Mental

For peer review only - http://bmjopen.bmj.com/site/about/guidelines.xhtml

State Examination, CFI, EQ-5D, Five Cog, Timed Up & Go test, Physical Activity Scale for the Elderly and Geriatric Depression Scale. In the analysis of variance between the three groups, effect size was set at 0.25, α=0.05, and power=0.80, the necessary data number will be approximately 50 persons for each group. Since this is a long-term longitudinal study, the number of subjects is set at a higher 100 persons, which is about double the necessary number (for statistical significance) as a cautionary measure for drop outs and missing data. The longitudinal change of the evaluation results in each group is analyzed using repeated analysis of variance and mixed effects model. The amount of change in the evaluation result between groups is compared by multiple comparison tests: the paired t-test or Wilcoxon test.

Discussion

In a prior study (Kojima et al, 2019) an analysis of 182,099 Kobe citizens showed, cognitively unfavorable answers in KCL were found to be associated with the incidence of LTC need, independent of age, sex and other items on KCL [15]. Furthermore, as the number of unfavorable answers to the cognitive domain increased from 0 to 1, 2 and 3, the incidence of LTC need progressively increased from 3.5% to 6.4%, 12.6%, and 29.6% after 3 years. This finding suggests a potential utility for KCL in the risk stratification of future LTC need. However, because this was a retrospective analysis on pooled data, the causal relationship between cognitively unfavorable answers and LTC need could not be validated. The current

For peer review only - http://bmjopen.bmj.com/site/about/guidelines.xhtml

project will test the predictive ability of the KCL as well as other tools such as CFI, EQ-5D and "Frail Kenshin" checkup in relation to LTC need.

Each of the 4 studies in this project focuses on citizens at a different level of dementia risk (Study 1: general population, Study 2: higher risk population, Study 3: lower risk population, Study 4: higher risk individuals willing to participate in the training program), see Table 1.

Strengths & weakness of the study

A limitation of the study is that the follow up study period is relatively short, due to the contract duration by the funder. However, if this evaluation system is incorporated into local governmental systems in the future, follow up periods can be virtually unlimited, and baseline and outcome evaluation would be repeated (Figure 1). Through this process a quantitative feedback mechanism could be created forming the foundation to reduce the social burden of dementia, which further on, could also be extended to address other public health problem areas [16,17]. Additionally, Study 4 is a highly exploratory study with limited sample size. This study hopes to find what is feasible and possibly provide clues for improving the social burden of LTC need, which can then be tested in future studies.

Meaning of study

By the completion of Study 1, 2 and 3, risk stratification of earlier cognitive decline for future

For peer review only - http://bmjopen.bmj.com/site/about/guidelines.xhtml

LTC need will be improved on a population basis. Particularly, if individuals at risk for future dementia could be extracted, social efforts could be directed to such individuals who may receive a greater benefit, allowing for a more efficient allocation of limited public resources. Also, given the failure of many clinical trials for dementia drug development, the system constructed by this project could help prepare "trial-ready cohorts", to increase efficiency of recruiting eligible participants.

In summary, this project hopes to show a risk prediction and early intervention model linked to the outcome measurement, LTC. If continuously implemented, it can be a foundation for addressing the burden of dementia earlier. The final hope of this project is "not to be demented", but if demented, "not to be in need of care".

Patient and public involvement

No Patient Involved.

Ethics and dissemination

This study complies with the Declaration of Helsinki. These studies were approved by the Ethics Review Committee of the World Health Organization (ERC.0002899) as well as respective organizational ERC's, and the Ethics Review Committee of the Kobe Municipal office. In addition, the study conforms to the provisions of the Declaration of Helsinki and are conducted in accordance with Japan's "Ethical Guidelines for Medical and Health Research Involving Human Subjects.

The researchers plan to disseminate results at conferences and in peer-reviewed journals.

For peer review only
For peer review only - http://bmjopen.bmj.com/site/about/guidelines.xhtml

References

1 Nishimura S. Social Security In Japan. Published Online First: March 2011.http://www.ipss.go.jp/s-info/e/Jasos2011/ss2011.pdf (accessed 31 Dec 2014).

2 The World Bank. Population ages 65 and above, total. World Bank Data. 2018.https://data.worldbank.org/indicator/SP.POP.65UP.TO.ZS?end=2019&locations=JP&start=2019 (accessed 22 Feb 2021).

3 Cabinet Office. Health and Welfare of the Elderly | The White Paper on the Aged Society in 2016 (The whole edition). 2020. http://www8.cao.go.jp/kourei/whitepaper/w-2016/html/zenbun/s1_2_3.html (accessed 22 Feb 2021).

4 World Health Organization - Alzheimer's Disease International. DEMENTIA: A Public Health Priority. Published Online First: 2012.http://apps.who.int/iris/bitstream/10665/75263/1/9789241564458_eng.pdf?ua=1 (accessed 6 Feb 2017).

5 Fukawa T. Prevalence of dementia among the elderly population in Japan. *Heal Prim Care* 2018;**2**. doi:10.15761/hpc.1000147

6 Rubinstein E, Duggan C, Landingham B Van, *et al.* A CALL TO ACTION THE GLOBAL RESPONSE TO DEMENTIA THROUGH POLICY INNOVATION. http://www.mhinnovation.net/sites/default/files/downloads/resource/WISH_Dementia_Forum_Report_08.01.15_WEB.pdf (accessed 3 Apr 2017).

7 UK Government. G8 dementia summit: Global action against dementia - 11 December 2013. Published Online First: 2013.https://www.gov.uk/government/publications/g8-dementia-summit-global-action-against-dementia/g8-dementia-summit-global-action-against-dementia-11-december-2013 (accessed 6 Feb 2017).

8 Imtiaz B, Tolppanen A-M, Kivipelto M, *et al.* Future directions in Alzheimer's disease from risk factors to prevention. *Biochem Pharmacol* 2014;**88**:661–70. doi:10.1016/j.bcp.2014.01.003

9 Urakami K. [Prevention of dementia]. *Nihon Rinsho* 2016;**74**:395–8.http://www.ncbi.nlm.nih.gov/pubmed/27025075 (accessed 2 Feb 2017).

For peer review only - http://bmjopen.bmj.com/site/about/guidelines.xhtml

10 Wu Y-T, Fratiglioni L, Matthews FE, *et al.* Dementia in western Europe: epidemiological evidence and implications for policy making. *Lancet Neurol* 2016;**15**:116–24. doi:10.1016/S1474-4422(15)00092-7

11 Nakanishi M, Nakashima T. Features of the Japanese national dementia strategy in comparison with international dementia policies: How should a national dementia policy interact with the public health- and social-care systems? *Alzheimer's Dement* 2014;**10**:468-476.e3. doi:10.1016/j.jalz.2013.06.005

12 Amariglio RE, Donohue MC, Marshall GA, *et al.* Tracking early decline in cognitive function in older individuals at risk for Alzheimer disease dementia: the Alzheimer's Disease Cooperative Study Cognitive Function Instrument. *JAMA Neurol* 2015;**72**:446–54. doi:10.1001/jamaneurol.2014.3375

13 Sampaio PYS, Sampaio RAC, Yamada M, *et al.* Systematic review of the Kihon Checklist: Is it a reliable assessment of frailty? *Geriatr Gerontol Int* 2016;**16**:893–902. doi:10.1111/ggi.12833

14 Satake S, Shimokata H, Senda K, *et al.* Validity of Total Kihon Checklist Score for Predicting the Incidence of 3-Year Dependency and Mortality in a Community-Dwelling Older Population. *J Am Med Dir Assoc* 2017;**18**:552.e1-552.e6. doi:10.1016/j.jamda.2017.03.013

15 Kojima S, Murotani K, Zhou B, *et al.* Assessing long-term care risk in older individuals with possible cognitive decline: A large population-based study using the Kihon Checklist. *Geriatr Gerontol Int* 2019;**19**. doi:10.1111/ggi.13677

16 Msanori Fukushima, Kenichi Kohno, Shinsuke Kojima, Keisuke Yuasa, Kenichiro Tanabe YN. For the sharp decrease in the social burden of medical expenses, nursing care - Design of the area data utilization hubs and the infrastructure development to provide curative treatment. *Clin Eval* 2019;**47**:105–15.http://cont.o.oo7.jp/47sup36/47sup36contents_e.html (accessed 8 Jul 2020).

17 Fukushima. M. The reality of 2040 issues in Japan - Building the learning health society as a solution. *Clin Eval (in Press)* 2020.

Contributorship Statement

YK, YN, SK, HK, YY, HK, TO & RK contributed to the conception and design of this research project and the four individual studies therein. YN, YK and KK participated in the

For peer review only - http://bmjopen.bmj.com/site/about/guidelines.xhtml

preparation of the manuscript, and all authors critically revised the manuscript for important intellectual content. YK is the corresponding author and YK and YN are the guarantors of the work. The corresponding author attests that all listed authors meet authorship criteria and that no others meeting the criteria have been omitted.

Acknowledgements

We express our sincere gratitude to Miyuki Kawabata for the overall project management, and Ayumi Okada for the data management of respective studies in this project. Also, we are deeply grateful for the robust corporation of Kobe city municipal office, including the data collection, processing and anonymization, as well as all other efforts for the planning and implementation of this project. Further we acknowledge the Alzheimer's Disease Cooperative Study for permission for the use of the CFI questionnaire. Used with permission from the NIA-funded Alzheimer's Disease Cooperative Study (NIA Grant U19 AG10483)

Funding

This work was funded by the_World Health Organization and is supported by the World Health Organization Centre for Health Development (WHO Kobe Centre) grant number WKC: K18001. ERC.0002899. the Sponsor provided funding, but had no role in the research project design, or preparation and submission of the manuscript. The researchers are independent from the funders and all authors, internal and external, had full access to all the data in the study and can take responsibility for the integrity of the data and the accuracy of the data analysis.

Conflicts of Interest

All authors have completed the ICMJE uniform disclosure form at www.icmje.org/coi_disclosure.pdf and declare: no support from any organization for the submitted work; no financial relationships with any organizations that might have an interest in the submitted work in the previous three years; no other relationships or activities that could appear to have influenced the submitted work.

For peer review only - http://bmjopen.bmj.com/site/about/guidelines.xhtml

Transparency declaration

Dr. Yasumasa Kakei affirms that the manuscript is honest, accurate, and a transparent account of the study being reported; no important aspects of the study has been omitted; any discrepancies from the study as originally planned have been explained.

Copyright/license for publication

The Corresponding Author has the right to grant on behalf of all authors and does grant on behalf of all authors, a worldwide license to the Publishers and its licensees in perpetuity, in all forms, formats and media (whether known now or created in the future), to i) publish, reproduce, distribute, display and store the Contribution, ii) translate the Contribution into other languages, create adaptations, reprints, include within collections and create summaries, extracts and/or, abstracts of the Contribution, iii) create any other derivative work(s) based on the Contribution, iv) to exploit all subsidiary rights in the Contribution, v) the inclusion of electronic links from the Contribution to third party material where-ever it may be located; and, vi) license any third party to do any or all of the above.

Figure & Table Legend:

Table 1: Summary of studies

Figure 1. Project Concept. This figure depicts the concept of this project. If baseline and outcome evaluation, can we repeated and incorporated into government practice, a foundation for the focus on high-risk individuals and possible reduction in LTC (social burden) can be realized.

For peer review only

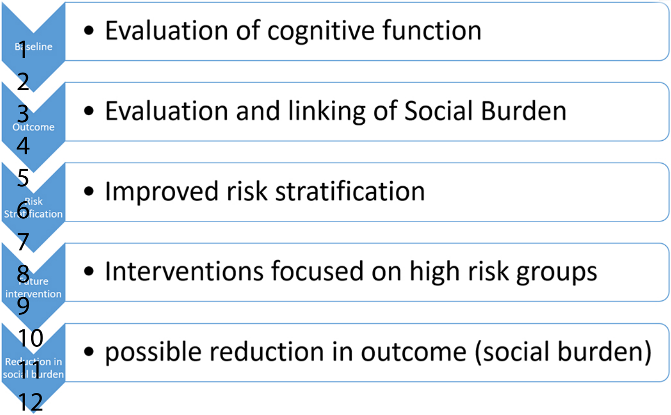

Figure 1. Project Concept. This figure depicts the concept of this project. If baseline and outcome evaluation can we repeated and incorporated into government practice, a foundation for the focus on high risk individuals and possible reduction in LTC (social burden) can be realized.

er review only - http://bmjopen.bmj.com/site/about/guidelines

Geriatrics & Gerontology INTERNATIONAL

Medline Indexed

RESEARCH STUDIES

English translation of the Kihon Checklist

Dear Editor,

Since the introduction of the long-term care insurance (LTCI) system in 2000, the number of older persons with LTCI service requirement has been increasing in Japan. Therefore, the Japanese Ministry of Health, Labor and Welfare started the Care Prevention Programs in 2006 to prevent frailty and disability of older persons, and introduced the “Kihon Checklist” (KCL) to identify vulnerable older adults as those at a higher risk of becoming dependent. The KCL has 25 yes/no questions divided into seven domains: activities of daily living, physical strength, nutrition, oral function, isolation, memory and mood, and has been used for screening frail older adults. Indeed, many studies have shown that the KCL was able to predict older persons who required the LTCI service and to assess frailty.[1–3] However, several reports used different English translations of the KCL in their manuscripts.[3–6] Additionally, Sampaio *et al.* created the Portuguese version of the KCL, and used it to assess frailty in Brazilian older adults, showing that the KCL can be used for elderly care in other countries as well.[4] Therefore, we decided to create the official version of the KCL.

The translation of the KCL into English was carried out by two people bilingual in Japanese and English. Next, the English version of KCL was back-translated into Japanese by one of the members of the Working Group on Frailty. This process was used to verify whether or not the KCL back-translation contained any incorrect expressions compared with the original Japanese version of the KCL. We then ensured the similarity of the contents between the original and back-translated versions of the KCL. After we approved the final version of the KCL, we asked a native English speaker to check the English (Table 1). We hope that this English version

For peer review only

Table 1 English translation of the Kihon Checklist

No.	Questions	Answer	
1	Do you go out by bus or train by yourself?	□0. YES	□1. NO
2	Do you go shopping to buy daily necessities by yourself?	□0. YES	□1. NO
3	Do you manage your own deposits and savings at the bank?	□0. YES	□1. NO
4	Do you sometimes visit your friends?	□0. YES	□1. NO
5	Do you turn to your family or friends for advice?	□0. YES	□1. NO
6	Do you normally climb stairs without using handrail or wall for support?	□0. YES	□1. NO
7	Do you normally stand up from a chair without any aids?	□0. YES	□1. NO
8	Do you normally walk continuously for 15 minutes?	□0. YES	□1. NO
9	Have you experienced a fall in the past year?	□1. YES	□0. NO
10	Do you have a fear of falling while walking?	□1. YES	□0. NO
11	Have you lost 2 kg or more in the past 6 months?	□1. YES	□0. NO
12	Height: cm, weight: kg, BMI: kg/m^2 If BMI is less than 18.5, this item is scored.	□1. YES	□0. NO
13	Do you have any difficulties eating tough foods compared to 6 months ago?	□1. YES	□0. NO
14	Have you choked on your tea or soup recently?	□1. YES	□0. NO
15	Do you often experience having a dry mouth?	□1. YES	□0. NO
16	Do you go out at least once a week?	□0. YES	□1. NO
17	Do you go out less frequently compared to last year?	□1. YES	□0. NO
18	Do your family or your friends point out your memory loss? e.g.“You ask the same question over and over again.”	□1. YES	□0. NO
19	Do you make a call by looking up phone numbers?	□0. YES	□1. NO
20	Do you find yourself not knowing today’s date?	□1. YES	□0. NO
21	In the last 2 weeks have you felt a lack of fulfilment in your daily life?	□1. YES	□0. NO
22	In the last 2 weeks have you felt a lack of joy when doing the things you used to enjoy?	□1. YES	□0. NO
23	In the last 2 weeks have you felt difficulty in doing what you could do easily before?	□1. YES	□0. NO
24	In the last 2 weeks have you felt helpless?	□1. YES	□0. NO
25	In the last 2 weeks have you felt tired without a reason?	□1. YES	□0. NO

Working Group on Frailty in Japanese Geriatrics Society. BMI, body mass index.

On behalf of the Working Group on Frailty in the Japan Geriatrics Society

 doi: 10.1111/ggi.12397 © 2015 Japan Geriatrics Society

For peer review only - http://bmjopen.bmj.com/site/about/guidelines.xhtml

of the KCL can be widely used for elderly care in other countries as well as Japan.

We thank Akemi Hamaya and Toshiko Yamashita for back-translation, and Kasia Malinowska, Kyoto University, for checking the final English version of the KCL.

Disclosure statement

The authors declare no conflict of interest.

Hidenori Arai[1] and Shosuke Satake[2]
[1]*Department of Human Health Sciences, Kyoto University Graduate School of Medicine, Kyoto, and* [2]*National Center for Geriatrics and Gerontology, Obu, Japan*

References

1 Nemoto M, Yabushita N, Kim M, Matsuo T, Seino S, Tanaka K. Assessment of vulnerable older adult's physical function according to the Japanese Long-Term Care Insurance (LTCI) system and Fried's criteria for frailty syndrome. *Arch Gerontol Geriatr* 2012; **55**: 385–391.
2 Tomata Y, Hozawa A, Ohmori-Matsuda K *et al*. Validation of the Kihon Checklist for predicting the risk of 1-year incident long-term care insurance certification: the Ohsaki Cohort 2006 Study. *Nippon Koshu Eisei Zasshi* 2011; **58**: 3–13.
3 Fukutomi E, Okumiya K, Wada T *et al*. Importance of cognitive assessments as part of the "Kihon Checklist" developed by the Japanese Ministry of Health, Labour and Welfare for prediction of frailty at a 2-year follow up. *Geriatr Gerontol Int* 2013; **13**: 654–662.
4 Sewo Sampaio PY, Sampaio RA, Yamada M, Ogita M, Arai H. Validation and translation of the Kihon Checklist (frailty index) into Brazilian Portuguese. *Geriatr Gerontol Int* 2014; **14**: 561–569.
5 Umegaki H, Suzuki Y, Yanagawa M, Nonogaki Z, Nakashima H, Endo H. Dysphagia in older adults at high risk of requiring care. *Geriatr Gerontol Int* 2012; **12**: 359–361.
6 Koizumi Y, Hamazaki Y, Okuro M *et al*. Association between hypertension status and the screening test for frailty in elderly community-dwelling Japanese. *Hypertens Res* 2013; **36**: 639–644.

Operationalization of a frailty index using routine data from the Toulouse Frailty Clinic

Dear Editor,

Frailty in older adults is defined as a geriatric syndrome characterized by the loss of reserve capacity in multiple physiological systems, associated with increased vulnerability to adverse outcomes.[1,2] A widely accepted instrument to measure frailty is Rockwood's frailty index (FI), which counts deficits in health (e.g. diseases, disabilities).[3,4] Very few studies have used clinical data from routine care to operationalize a FI.[5] Therefore, in the current study, routine data from a geriatric day-hospital unit in France were used to create a FI. We studied the distribution of this FI, and we investigated age-related patterns in the FI score.

Cross-sectional data were used from frail older patients admitted to the Toulouse Frailty Clinic in France, a geriatric day hospital unit of the Toulouse University Hospital. Community dwelling frail older persons (identified by their general practitioner as frail using the Gerontopole Frailty Screening Tool) are referred to this day hospital for undergoing a comprehensive geriatric assessment.[6] Details of the assessment have been described elsewhere.[7] Of 488 patients admitted between 1 January and 1 November in 2013, 484 were included. A 35-item FI was constructed, with items on chronic diseases, basic and instrumental disabilities, serum vitamin D, cognition, physical performance, obesity, visual and hearing impairment, and malnutrition.[8] The FI score indicates the proportion of deficits present (range 0–1). The FI score was calculated for all 484 patients with the recommended minimum of 30 items (just 7.8% had between 1 to 5 missing items). Descriptive statistics were used to study the distribution of the FI. Linear regression analyses were carried out to test linear and non-linear associations between age and the FI score (SPSS 20; IBM, Armonk, NY, USA).

The mean age of the 484 included patients was 83.2 years (SD 6.0; range 65–103 years), and there were 305 women (63.0%). The mean FI score was 0.32 (SD 0.14; men 0.33, SD 0.14; women 0.31, SD 0.13). The distribution of the FI ranged from 0.09 to 0.83 with a 99% upper limit of 0.67 (Fig. 1a). The FI score was significantly associated with age (increase in mean FI score of 0.009 per year, 95% CI 0.007–0.011; $P < 0.001$). The association seemed stronger at more advanced ages (Fig. 1b; quadratic term for age $P < 0.01$).

The distribution of the FI created with routine data from a geriatric day hospital was quite similar compared with previous work using research data.[3,8] Within a population of moderate to severe frail older patients, the constructed FI was able to produce a heterogeneous range of results, which implies a more subtle

© 2015 Japan Geriatrics Society
doi: 10.1111/ggi.12403

For peer review only - http://bmjopen.bmj.com/site/about/guidelines.xhtml

Site: ________________

Participant Number: ________________

Cognitive Function Index

Participant

Individual Overseeing Completion:

Initials: ________ Date: ____________

Instructions: Please complete this form independently, without consulting anyone. Answer all questions with reference to one year ago.

1. Compared to one year ago, do you feel that your memory has declined substantially?

   ☐ **Yes** ☐ **No** ☐ **Maybe**

2. Do others tell you that you tend to repeat question over and over?

   ☐ **Yes** ☐ **No** ☐ **Maybe**

3. Have you been misplacing things more often?

   ☐ **Yes** ☐ **No** ☐ **Maybe**

4. Compared to one year ago, do you find that you are relying more on written reminders (e.g., shopping lists, calendars)?

   ☐ **Yes** ☐ **No** ☐ **Maybe**

5. Do you need more help from others to remember appointments, family occasions or holidays?

   ☐ **Yes** ☐ **No** ☐ **Maybe**

6. Do you have more trouble recalling names, finding the right word, or completing sentences?

   ☐ **Yes** ☐ **No** ☐ **Maybe**

7. Do you have more trouble driving (e.g., do you drive more slowly or too fast, have more trouble at night, tend to get lost, have accidents)?

   ☐ **Yes** ☐ **No** ☐ **Maybe** ☐ **Does Not Apply**

8. Compared to one year ago, do you have more difficulty managing money (e.g., paying bills, calculating change, completing tax forms)?

   ☐ **Yes** ☐ **No** ☐ **Maybe** ☐ **Does Not Apply**

9. Are you less involved in social activities?

   ☐ **Yes** ☐ **No** ☐ **Maybe**

10. Has your work performance (paid or volunteer) declined significantly, compared to one year ago?

    ☐ **Yes** ☐ **No** ☐ **Maybe** ☐ **Does Not Apply**

For peer review only

For peer review only - http://bmjopen.bmj.com/site/about/guidelines.xhtml

Site: _______________

Participant Number: _______________

Cognitive Function Index

Participant

Individual Overseeing Completion:

Initials: _______ Date: ____________

11. Do you have more trouble following the news or the plots of books, movies, or TV shows?

☐ Yes ☐ No ☐ Maybe

12. Are there any activities (e.g., hobbies such as card games, crafts) that are substantially more difficult for you now compared to one year ago?

☐ Yes ☐ No ☐ Maybe

13. Are you more likely to be come disoriented, or get lost, for example when traveling to another city?

☐ Yes ☐ No ☐ Maybe

14. Compared to one year ago, do you have more difficulty using household appliances (such as washing machine or microwave) or electronic devices (such as computer or cell phone)?

☐ Yes ☐ No ☐ Maybe

15. In the past year, have you seen a doctor about memory concerns?

☐ Yes ☐ No

For peer review only

To be completed by study staff:

**Method of Collection**	**Comments:**
___ In-person/Self<br>___ By phone/Interview	______<br>______<br>______

For peer review only - http://bmjopen.bmj.com/site/about/guidelines.xhtml

EQ-5D-5L

健康アンケート

日本用日本語版

(Japanese version for Japan)

For peer review only

Japan (Japanese) © 2009 EuroQol Group. EQ-5D™ is a trade mark of the EuroQol Group

各項目において、あなたの**今日の**健康状態を最も良く表している四角（❑）**1つ**に✓印をつけてください。

移動の程度

歩き回るのに問題はない ❑

歩き回るのに少し問題がある ❑

歩き回るのに中程度の問題がある ❑

歩き回るのにかなり問題がある ❑

歩き回ることができない ❑

身の回りの管理

自分で身体を洗ったり着替えをするのに問題はない ❑

自分で身体を洗ったり着替えをするのに少し問題がある ❑

自分で身体を洗ったり着替えをするのに中程度の問題がある ❑

自分で身体を洗ったり着替えをするのにかなり問題がある ❑

自分で身体を洗ったり着替えをすることができない ❑

ふだんの活動 *（例：仕事、勉強、家事、家族・余暇活動）*

ふだんの活動を行うのに問題はない ❑

ふだんの活動を行うのに少し問題がある ❑

ふだんの活動を行うのに中程度の問題がある ❑

ふだんの活動を行うのにかなり問題がある ❑

ふだんの活動を行うことができない ❑

痛み ／ 不快感

痛みや不快感はない ❑

少し痛みや不快感がある ❑

中程度の痛みや不快感がある ❑

かなりの痛みや不快感がある ❑

極度の痛みや不快感がある ❑

不安 ／ ふさぎ込み

不安でもふさぎ込んでもいない ❑

少し不安あるいはふさぎ込んでいる ❑

中程度に不安あるいはふさぎ込んでいる ❑

かなり不安あるいはふさぎ込んでいる ❑

極度に不安あるいはふさぎ込んでいる ❑

Japan (Japanese) © 2009 EuroQol Group. EQ-5D™ is a trade mark of the EuroQol Group

For peer review only - http://bmjopen.bmj.com/site/about/guidelines.xhtml

For peer review only

- あなたの**今日の**健康状態がどのくらい良いか悪いかを教えてください。
- このものさしには **0** から **100** までの目盛がふってあります。
- **100** はあなたの想像できる最も良い健康状態を、

  0 はあなたの想像できる最も悪い健康状態を表しています。
- **今日の**健康状態がどのくらい良いか悪いかを、このものさし上に×印をつけて表してください。
- ものさし上に×印をつけたところの目盛を下の四角に記入してください。

あなたの今日の健康状態 ＝ ☐

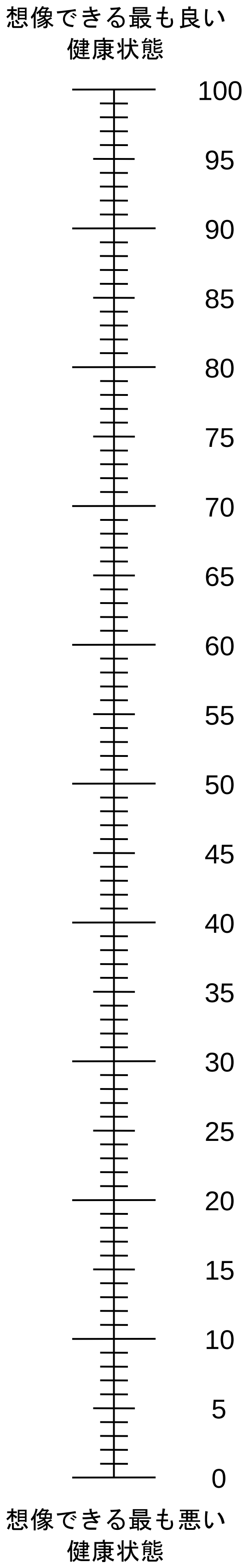

Japan (Japanese) © 2009 EuroQol Group. EQ-5D™ is a trade mark of the EuroQol Group

For peer review only - http://bmjopen.bmj.com/site/about/guidelines.xhtml

神戸市　「フレイル健診」試行実施　計測（運動・筋力、咀嚼力、嚥下）結果記入票

下記の太枠内をご記入ください。

氏名　　　　　　　男・女	生年月日　　　　昭和　　年　　月　　日　（　　）才<br>神戸市国民健康保険 被保険者番号：受診券・健康保険証に記載あり（　　　　　　　）
1．【ふくらはぎ周囲長】<br>（　　　　　）ｃｍ<br>男性は 34ｃｍ未満、女性は 32ｃｍ未満の場合は、要注意です。	2．【指輪っかテスト】　（　太くて囲めない　・　ちょうど囲める　・　隙間ができる　）<br>＊「指輪っかテスト」は、東京大学社会総合研究機構で考案されたものです。<br>「隙間ができる」に当てはまる場合には、筋肉や筋量が減っている可能性があります。<br>ご自分で筋肉の低下を測る目安となります。筋力の衰えが気になる時に測定してみましょう。

3．【かむ力判定ガム】　　緑　・　黄　・　桜色　・　濃い桜色　・　紅色

＜咀嚼力＞　弱い

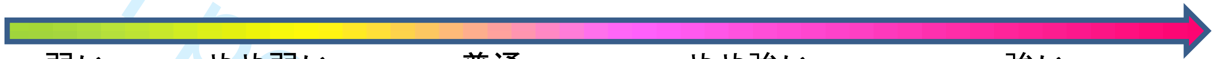

強い

弱い　　やや弱い　　普通　　やや強い　　強い

一口（ひとくち）30 回よくかんで食べること（カミング３０）は、全身の健康や認知症予防に大きな効果があります。かめない場合は、かかりつけ歯科医を受診しましょう。

4．【だ液ゴックンテスト】　30 秒の間に唾液を飲み込んだ回数　（2 回以下・3 回以上）　3 回以上飲み込めれば、問題はありません。

結果		[illegible]<br>＊握力が強い方の手の握力					[illegible] 15[illegible]				
性別・年代		弱い	やや弱い	ふつう	やや強い	強い	遅い	やや遅い	ふつう	やや早い	早い
男性	65－69	～24kg	24.1～31.0	31.1～34.0	34.1～43.0	43.1～	～4回	5～7	8	9～11	12以上
	70－74	～22kg	22.1～29.0	29.1～32.0	32.1～40.0	40.1～	～4回	5～6	7	8～10	11以上
女性	65－69	～15kg	15.1～19.0	19.1～21.0	21.1～27.0	27.1～	～4回	5～7	8	9～11	12以上
	70－74	～14kg	14.1～18.0	18.1～20.0	20.1～26.0	26.1～	～4回	5～6	7	8～10	11以上
取り入れたい運動		握力は全身の筋力の状況と比例していて全身の体力の把握に役立ちます。「弱い」、「やや弱い」の方は生活に体操や筋力トレーニングを取り入れましょう。筋力が向上すると転倒予防になり、いろいろな動作がしやすくなります。					立ち上がり動作は足腰の筋力の強さや持久力・スタミナの目安になります。「遅い」、「やや遅い」の方は、散歩・ジョギング・ハイキングなどを取り入れましょう。坂道や階段の昇り降りが楽になり、楽に歩ける距離が長くなります。				

For peer review only

別紙2

氏名　　　　　　　　　　男・女	電話番号	
〒　　　　住所　　　　　　　区		
生年月日　昭和　　年　　月　　日（　　）才	神戸市国民健康保険　被保険者番号 ＊受診券・健康保険証に記載あり（　　　　）	

神戸市「フレイル健診」試行実施　受診票・質問票

この健診結果は、受診者本人に対して通知するとともに、神戸市において保存し、必要に応じて介護予防のためのご案内や、個人が特定されない方法で統計・調査研究などに活用しますので、ご了承の上、ご受診願います。

受診日　　年　　月　　日　　受診会場名

以下の太枠内を、ご記入ください。

1	この１年間に特定健診などの健診を受けましたか。	はい	いいえ

日常生活活動・もの忘れチェック

２．下記の質問にお答えください。

①	バスや電車で一人で外出していますか。	はい	いいえ
②	日用品の買い物をしていますか。	はい	いいえ
③	預貯金の出し入れをしていますか。	はい	いいえ
④	友人の家を訪ねていますか。	はい	いいえ
⑤	家族や友人の相談にのっていますか。	はい	いいえ
⑥	週に１回以上は外出していますか。	はい	いいえ
⑦	昨年と比べて外出の回数が減っていますか。	はい	いいえ
⑧	周りの人から「いつも同じ事を聞く」などのもの忘れがあると言われますか。	はい	いいえ
⑨	自分で電話番号を調べて、電話をかけることをしていますか。	はい	いいえ
⑩	今日が何月何日かわからない時がありますか。	はい	いいえ

運動機能チェック

３．下記の質問にお答えください。

①	階段を手すりや壁をつたわらずに昇っていますか。	はい	いいえ
②	椅子に座った状態から何もつかまらずに立ち上がっていますか。	はい	いいえ
③	15 分続けて歩いていますか。	はい	いいえ
④	この１年間に転んだことがありますか。	はい	いいえ
⑤	転倒に対する不安は大きいですか。	はい	いいえ

栄養状態チェック

４．下記の質問にお答えください。

①	６ヶ月間で２～３ｋｇ以上の体重減少がありましたか。	はい	いいえ
②	身長　　．　　ｍ　　体重　　　ｋｇ	―	―

口腔機能チェック

５．現在、ご自身の歯は何本残っていますか。当てはまる本数に○を付けてください。さし歯や金属をかぶせた歯は自分の歯に含めます。成人の歯の総本数は、親知らずを含めて 32 本です。

①．０本　②．１～４本　③．５～９本　④．10～19 本　⑤．20 本以上

６．次の ２５種類の食品について、噛めるものに○、噛みづらいものに△、噛めないものに×をつけてください。

①バナナ		②茹でたキャベツ		③煮たにんじん		④煮たさといも	
⑤煮たたまねぎ		⑥いちご		⑦ハム		⑧かまぼこ	
⑨佃煮昆布		⑩こんにゃく		⑪鳥のからあげ		⑫ローストチキン	
⑬リンゴ		⑭なすの漬物		⑮生キャベツ		⑯焼いた豚肉	
⑰大根の漬物		⑱あられ		⑲ピーナッツ		⑳いか刺身	
㉑生のにんじん		㉒酢だこ		㉓するめ		㉔干しいも	
㉕かた焼きせんべい							
「○」　　個			「△」　　個			「×」　　個	

７．下記の質問にお答えください。

①	半年前に比べて固いものが食べにくくなりましたか。	はい	いいえ
②	お茶や汁物等でむせることがありますか。	はい	いいえ
③	口の渇きが気になりますか。	はい	いいえ

こころの健康チェック

８．下記の質問にお答えください。

①	（ここ２週間）毎日の生活に充実感がない。	はい	いいえ
②	（ここ２週間）これまで楽しんでやれていたことが楽しめなくなった。	はい	いいえ
③	（ここ２週間）以前は楽にできていたことが今はおっくうに感じられる。	はい	いいえ
④	（ここ２週間）自分が役に立つ人間だとは思えない。	はい	いいえ
⑤	（ここ２週間）わけもなく疲れたような感じがする。	はい	いいえ

実施機関処理欄

For peer review only
