# Supplementary material for: Kobe project for the exploration of newer strategies to reduce the social burden of dementia: a study protocol of cohort and intervention studies
Source: BMJ Open. 2021 Jun 17;11(6):e050948. doi: 10.1136/bmjopen-2021-050948 (PMC8215256; doi:10.1136/bmjopen-2021-050948)
Supplement: Reviewer comments [file bmjopen-2021-050948.reviewer_comments.pdf]

PEER REVIEW HISTORY

BMJ Open publishes all reviews undertaken for accepted manuscripts. Reviewers are asked to complete a checklist review form (**http://bmjopen.bmj.com/site/about/resources/checklist.pdf**) and are provided with free text boxes to elaborate on their assessment. These free text comments are reproduced below.

ARTICLE DETAILS

**TITLE (PROVISIONAL)**	Kobe Project for the Exploration of Newer Strategies to Reduce the Social Burden of Dementia: A study protocol of cohort and intervention studies.
**AUTHORS**	Kakei, Yasumasa; Nagai, Yoji; Kojima, Shunsuke; Kowa, Hisatomo; Yamamoto, Yasuji; Kajita, Hiroyuki; Osaki, Tohmi; Kothari, Kavita; Kayano, Ryoma

This article was not externally reviewed at BMJ Open. Protocols that have been independently assessed prior to submission to BMJ Open are usually fast-tracked to publication on the grounds that further substantial changes will not be possible. This independent assessment will usually be external, independent review for both a substantial grant award from a non-commercial or government funder and ethics approval.
